# Light spectra of biophilic LED-sourced system modify essential oils composition and plant morphology of *Mentha piperita* L. and *Ocimum basilicum* L

**DOI:** 10.3389/fpls.2023.1093883

**Published:** 2023-01-19

**Authors:** Peter Beatrice, Gabriella Saviano, Marcella Reguzzoni, Fabio Divino, Francesca Fantasma, Donato Chiatante, Antonio Montagnoli

**Affiliations:** ^1^ Department of Biotechnology and Life Sciences, University of Insubria, Varese, Italy; ^2^ Department of Biosciences and Territory, University of Molise, Pesche, Italy; ^3^ Department of Medicine and Surgery, University of Insubria, Varese, Italy

**Keywords:** light emitting diode, biophilia, aromatic plants, plants secondary metabolites, GC-MS, CoeLux^®^, technological advantages, light spectrum

## Abstract

Investigating morphological and molecular mechanisms that plants adopt in response to artificial biophilic lighting is crucial for implementing biophilic approaches in indoor environments. Also, studying the essential oils (EOs) composition in aromatic plants can help unveil the light influence on plant metabolism and open new investigative routes devoted to producing valuable molecules for human health and commercial applications. We assessed the growth performance and the EOs composition of *Mentha x piperita* and *Ocimum basilicum* grown under an innovative artificial biophilic lighting system (CoeLux^®^), that enables the simulation of natural sunlight with a realistic sun perception, and compared it to high-pressure sodium lamps (control) We found that plants grown under the CoeLux^®^ light type experienced a general suppression of both above and belowground biomass, a high leaf area, and a lower leaf thickness, which might be related to the shade avoidance syndrome. The secondary metabolites composition in the plants’ essential oils was scarcely affected by both light intensity and spectral composition of the CoeLux^®^ light type, as similarities above 80% were observed with respect to the control light treatments and within both plant species. The major differences were detected with respect to the EOs extracted from plants grown under natural sunlight (52% similarity in *M. piperita* and 75% in *O. basilicum*). Overall, it can be speculated that the growth of these two aromatic plants under the CoeLux^®^ lighting systems is a feasible strategy to improve biophilic approaches in closed environments that include both plants and artificial sunlight. Among the two plant species analyzed, *O. basilicum* showed an overall better performance in terms of both morphological traits and essential oil composition. To increase biomass production and enhance the EOs quality (e.g., higher menthol concentrations), further studies should focus on technical solutions to raise the light intensity irradiating plants during their growth under the CoeLux^®^ lighting systems.

## Introduction

In recent years, biophilic approaches are gaining increasing attention as a philosophy that encourages the use of natural elements in the design of built environments (Keller et al., 2008) to provide human beings with greater contact with nature (Gillis and Gatersleben, 2015). The biophilia hypothesis proposes that humans have an innate connection with the natural world (Wilson, 1984) and that a shortage in human exposure to nature can lead to a significant reduction in health, well-being and performance (Hähn et al., 2020). Varying design strategies can provide direct (e.g. light, air, water, plants, etc.) or indirect experiences of nature (e.g. images of nature; natural material, colors, and shapes; simulating natural light and air; etc.) (Kellert and Calabrese, 2015). Among these different environmental features, plants and the light irradiating them often play a pivotal role in indoor biophilic design strategies. Numerous studies demonstrated that introducing plants into offices can have significant positive effects on attention, creativity, and productivity perceived by the occupants (Hähn et al., 2020), reducing anxiety and nervousness (Chang and Chen, 2005). Furthermore, window views were demonstrated to further boost these positive effects (Farley and Veitch, 2001). In particular, several properties of windows were reported to provide psychological and physiological positive effects on humans: providing a view to the outside, supporting knowledge of the weather and time of the day, regulating air quality, reducing feelings of claustrophobia (Canazei et al., 2016), and reducing monotony or boredom (Stone and Irvine, 1994). However, direct access to windows may not be possible in every design situation, such as in underground or specific medical environments. In these contexts, the use of innovative lighting systems may enable the simulation of natural sunlight and, thus, be of help for enhancing the biophilic approach. In this regard, a lighting system named CoeLux^®^, able to artificially reproduce sunlight, is already commercially available and can generate positive long-term psycho-physiological effects on humans as its actual counterpart (Canazei et al., 2016). The CoeLux^®^ lighting system, recently developed by a team of Italian physicists (Di Trapani and Magatti, 2014), is a LED-based technology that uses nanostructured materials and optical systems to reproduce the visual effect of the sun in a blue sky and project realistic shadows in the room, providing a real impression of natural sunlight together with all its properties (Di Trapani and Magatti, 2017). Several details that imitate nature can be noted: the sky is perceived with an indefinite depth, imitating the outdoor conditions of a cloudless day; the sun is perceived with the dimensions of the real counterpart and appears to move across the sky when the observer walks under the skylight; the shadows have a blue tone and change in color with the distance of the shadowing object. Watching these artificial skylights, the correctly dimensioned phenomena that occur immediately trick the brain and the light is perceived as coming from outdoors (Canazei et al., 2016). We have recently investigated the impact of the CoeLux^®^ lighting systems on plant growth using the model plant *Arabidopsis thaliana* (Beatrice et al., 2021) (Beatrice et al., 2022). The *A. thaliana* phenotype was characterized by low biomass production, low leaf area and low lamina-to-petiole ratio, suggesting the onset of a strong shade avoidance syndrome (SAS). Both spectral composition and low light intensity seem to be involved in these responses.

The growth and development of aromatic plants under the light irradiated by the CoeLux^®^ lighting systems could boost the positive biophilic effects of these artificial skylights in indoor environments, especially when these lighting systems are installed in kitchens or dining rooms. However, the knowledge regarding how plants can grow and adapt to this light type is still poor. Aromatic plants can secrete through specialized cells (i.e., trichomes) important secondary metabolites, called essential oils (Rehman and Asif Hanif, 2016), which are odorous, volatile, hydrophobic, highly concentrated and composed of a high variety of compounds, including monoterpenoids, sesquiterpenoids, and phenylpropanoids. Of the tens of compounds that compose essential oils, even trace constituents may provide a considerable contribution to altering the odor, flavor, and bioactivity of the oil (Samarth et al., 2017). During plant life, different factors can modify the composition of the produced oil (Lawrence, 2007). Among these, the quality of light is known to affect the essential oil production of aromatic plants, both in terms of oil yield (Mulas et al., 2006) and oil composition (Noguchi and Amaki, 2016). Further sources of variability can also arise from the light intensity (Tabbert et al., 2022), photoperiod length, seasonal and climatic variations, plant ontogeny, and abiotic stresses like water deficiency, soil salinity, and high temperatures (Sangwan et al., 2001). The manipulation of some of these parameters under controlled conditions (e.g., light spectrum and intensity) can be considered as a strategy to modulate the metabolic pathway of aromatic plants, shaping their essential oils content, and improving the quality of the final product.

In this study, we used the aromatic plants *Ocimum basilicum* (basil) and *Mentha x piperita* (mint) with a double aim: (i) to assess if these aromatic plant species can grow in indoor biophilic environments illuminated by the CoeLux^®^ lighting system and (ii) to investigate the effects of the CoeLux^®^ light spectra on the essential oil composition of these plants. Also, investigating the oil composition of these plants can help to further unveil the light quality and quantity influence on the plant metabolism and open new routes of investigation to produce specific molecules that are needed for human health or commercial applications. In accordance with the previous studies on *A. thaliana*, we hypothesized to observe reduced plant growth under the CoeLux^®^ light type, due to both the low light intensity and the light spectra of these lighting systems. Still, we expect to observe variations in the composition of the major and minor components of the essential oils, due to the activation of diverse metabolic pathways.

## Materials and methods

### Plant material and growth conditions

Seeds of *Ocimum basilicum* L. of the variety “Italian classic basil” were obtained from a commercial nursery (Sementi Dotto – Italy) and sown in 2 L cylindrical pots (height 14 cm, lower Ø 11 cm, and upper Ø 16 cm) filled with a commercial sterilized soil-less substrate (mixture of peat, siliceous sand, and bark humus 1:2:1). Plants were then grown for 30 days in a growth chamber equipped with high-pressure sodium (HPS) lamps, at a soil level light intensity of 200 µmol m^-2^s^-1^, and a photoperiod of 14 h (DLI = 10.08 mol m^-2^day^-1^). The temperature was maintained as close as possible to 22°C, with an air humidity ranging between 50% and 70%, and watering till saturation with tap water. After this initial growth period, the plants were subjected to light treatments as described below. *Mentha x piperita* L. plants were obtained from a commercial nursery (Maison aromatique – Italy). Young shoots were separated from the rhizome and transplanted to 2 L pots filled with sterilized soil-less substrate. The new plants were grown for 15 days under the same growth conditions used for *O. basilicum* before being subjected to different light treatments.

### Light treatments and experimental design

After the initial growth period, we subjected our plants to three different light treatments (LTs), respectively named HPS200, HPS100, and CoeLux^®^. For each LT and each species, 10 plants were grown for 38 days and subsequently sampled to perform morphological and essential oils analysis. Moreover, after the same initial growth conditions and period, ten additional plants of both species were moved outdoors under direct sunlight (SUN LT) as a natural reference for the composition of the essential oils only. For the CoeLux^®^ LT, plants were grown inside the sunbeam of this artificial sunlight. We used the highest light intensity achievable with this lighting technology, corresponding to 100 µmol m^-2^s^-1^ (DLI = 5.04 mol m^-2^day^-1^) at the soil level and up to 120-140 µmol m^-2^s^-1^ (DLI = 6.05-7.06 mol m^-2^day^-1^) at the top of the plants. The soil level had a distance of from the lighting system of 50 cm, while the top of the plants reached a 10 cm distance. The photoperiod was maintained at 14h as set during the initial seedlings’ growth. We chose HPS lamps as the control light type since they are historically considered ideal light sources for indoor plant growth (Islam et al., 2012) (Pinho et al., 2012). Two different light intensities were used, 100 µmol m^-2^s^-1^ (DLI = 5.04 mol m^-2^day^-1^; named HPS100 LT) and 200 µmol m^-2^s^-1^ (DLI = 10.08 mol m^-2^day^-1^; named HPS200 LT), both measured at the soil level. For these treatments, the light reached an intensity at the top of the plants of 120-140 µmol m^-2^s^-1^ (DLI = 6.05-7.06 mol m^-2^day^-1^) for the HPS100 LT and 240-280 µmol m^-2^s^-1^ (DLI = 12.10-14.11 mol m^-2^day^-1^) for the HPS200 LT. This setting was in line with the indoor production of leafy vegetables and herbs since the existing literature often reports values ranging from 100 µmol m^-2^s^-1^ to 300 µmol m^-2^s^-1^ (Pennisi et al., 2020; Balázs et al., 2022). In addition, the 100 µmol m^-2^s^-1^ light intensity used in the HPS100 LT corresponds to the maximum light intensity achievable under the CoeLux^®^ lighting systems. The 200 µmol m^-2^s^-1^ light intensity used in the HPS200 LT was chosen as a further control for assessing the sole effect of the light intensity within the same light spectra. Plants moved under direct sunlight were grown in an open field at the University of Insubria - Campus Bizzozero (45°47’53.4” N 8°51’17” E – 392m asl - Varese, Italy) and harvested during the balsamic period (July 2021). In the months of June and July, this region is characterized by an average daily light integral (DLI) of 50-55 mol m^-2^day^-1^ (ENEA, 2020) and a photoperiod of 14.8-15.7 h.

**Figure 1:**
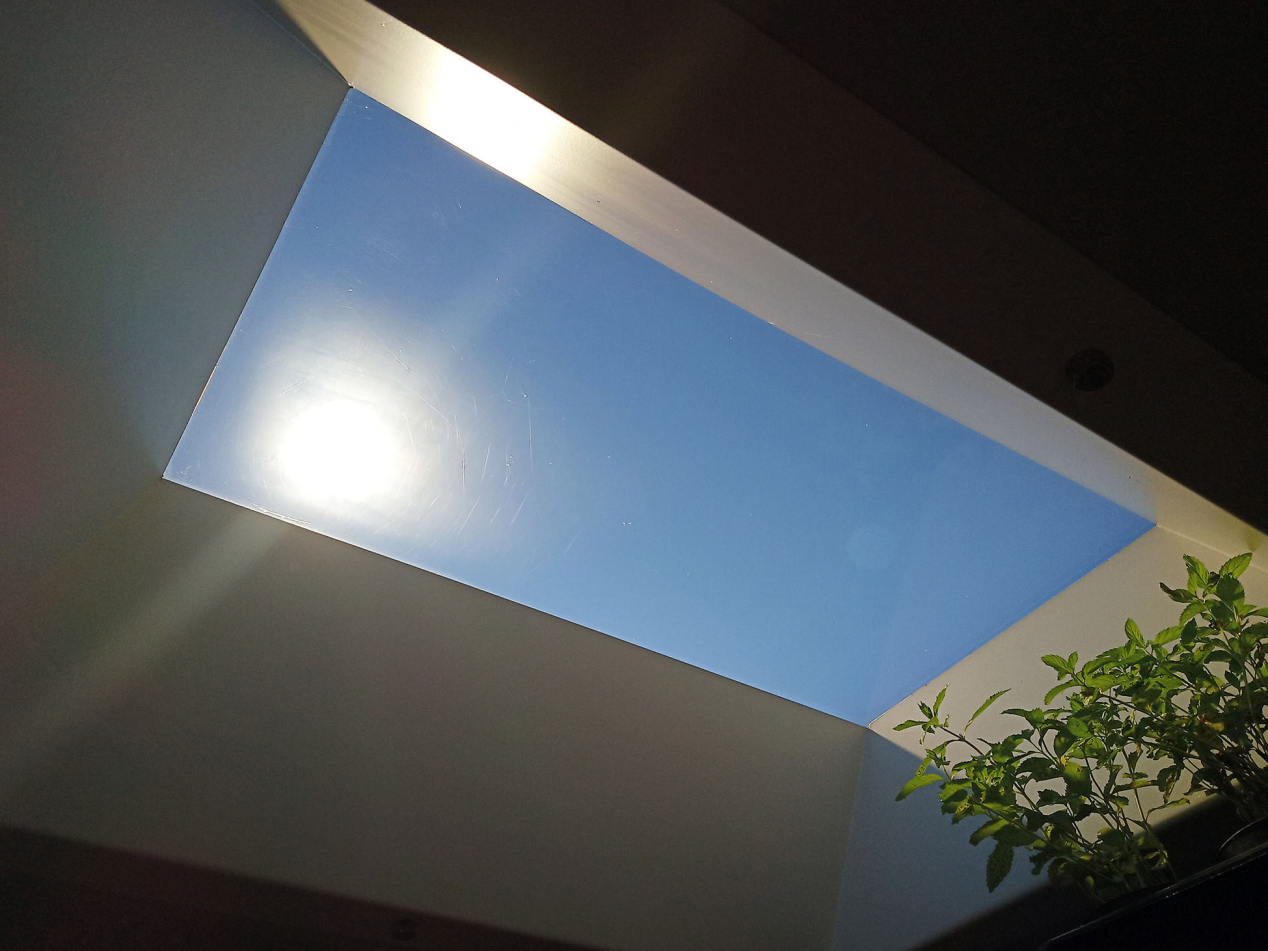
The visual appearance of the CoeLux^®^ lighting systems.

**Figure 2:**
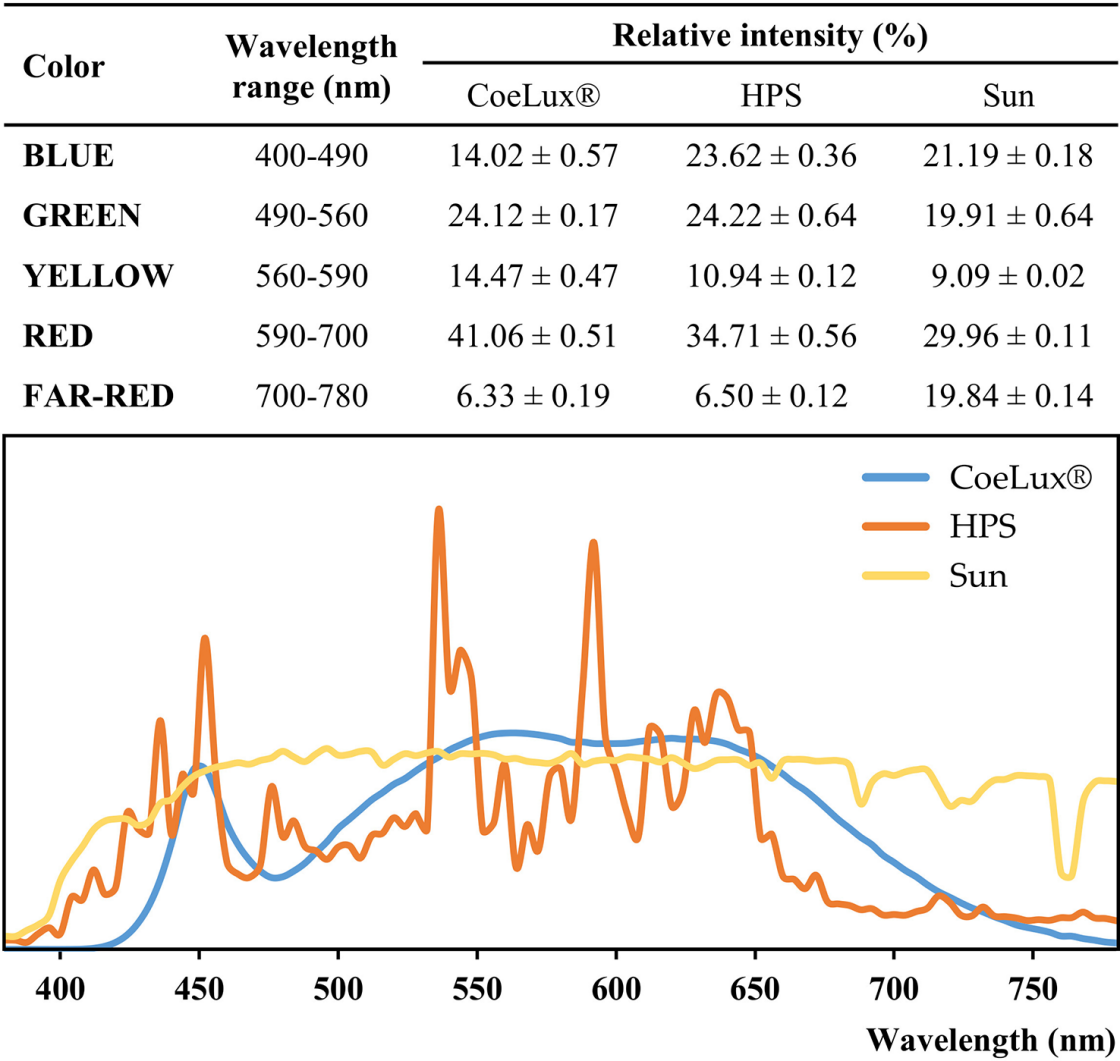
Spectra color composition and spectra curves of the CoeLux^®^ (blue),the HPS (orange), and the Sun (yellow) light types. Spectra measurements were collected in the range between 380 nm and 780 nm for each light type. To allow the comparison, photon counts measurements were normalized on the luminance of the respective spectrum. In the table, the sum of normalized photon counts was calculated for each color interval and reported in the form of relative intensity ± standard deviation. Spectra curves represent the mean of 8 different measurements (n=8).

The CoeLux^®^ lighting systems are engineered to perfectly resemble the natural light and sun appearance (Di Trapani and Magatti, 2014); the light is sourced by full-spectrum white LEDs, subsequently filtered to obtain the desired natural effect (**Figure 1**) (Di Trapani and Magatti, 2017). Both the CoeLux^®^ and HPS light types were characterized in a previous study (Beatrice et al., 2021), using the HD 2302.0 Light Meter (Delta Ohm) to measure the light intensity and the SpectraScan PR655 (Photo Research) to measure the spectra every 4 nm in the range between 380 nm and 780 nm (**Figure 2**). Spectra measurements were divided into color components, blue is the integral between 400-490 nm, green is the integral between 490-560 nm, yellow is the integral between 560-590 nm, red is the integral between 590-700 nm, and far-red is the integral between 700-780 nm. To calculate the red-to-far-red ratio (R/FR), red and far-red light was integrated between 650 and 670 nm and between 720 and 740 nm, while to calculate the blue-to-green ratio (B/G), blue and green light were integrated between 420 and 490 nm and between 500 and 570 nm, according to (Sellaro et al., 2010). Statistically significant differences were assessed using the *post hoc* Dunnett’s test. The HPS light type has a higher blue component, while the CoeLux^®^ light type has more yellow and red components. Green and far-red components showed no statistically significant differences. Despite similar values of FR light, the R/FR is higher under the CoeLux^®^ light type (4.68 ± 0.07) compared to the HPS light type (2.43 ± 0.02). While the B/G is higher under the HPS light type (0.83 ± 0.02) rather than under the CoeLux^®^ light type (0.50 ± 0.02). Additional spectra measurements were collected during a cloudless day in the location where the plants subjected to the SUN LT were grown. With respect to natural sunlight and considering equal light intensities, we observed a higher green, yellow, and red component in the CoeLux^®^ and HPS light types. The blue component was higher in the HPS light type and lower in the CoeLux^®^ light type, while the SUN light type had significantly higher levels of far-red. Under natural sunlight, we calculated an R/FR of 2.44 ± 0.02 and a B/G of 0.82 ± 0.02.

### Morphological analysis

All leaves were detached from the stems and both organs were separately scanned at 800 dpi with the Epson Expression 12000XL instrument and then oven-dried at 70°C until constant weight. The roots were freed from the soil media by carefully washing them under running water and subsequently oven-dried at 70°C until constant weight. The dry organs were then weighed separately on an analytical scale (Orma AL220S) to obtain the leaves, shoot (leaves + stem), and root biomass (LB, SB, and RB) and calculate the shoot-to-root ratio (S/R). The scanned images were processed with WinRhizo (Regent Instrument Inc., Quebec, Canada) to measure the leaf area (LA) and with ImageJ software (National Institute of Health, USA) to measure the lamina and petiole length of three leaves for each plant. Subsequently, the leaf mass per area (LMA) and the lamina-to-petiole length ratio (L/P) were calculated.

### Leaf anatomical analysis

**Figure 3:**
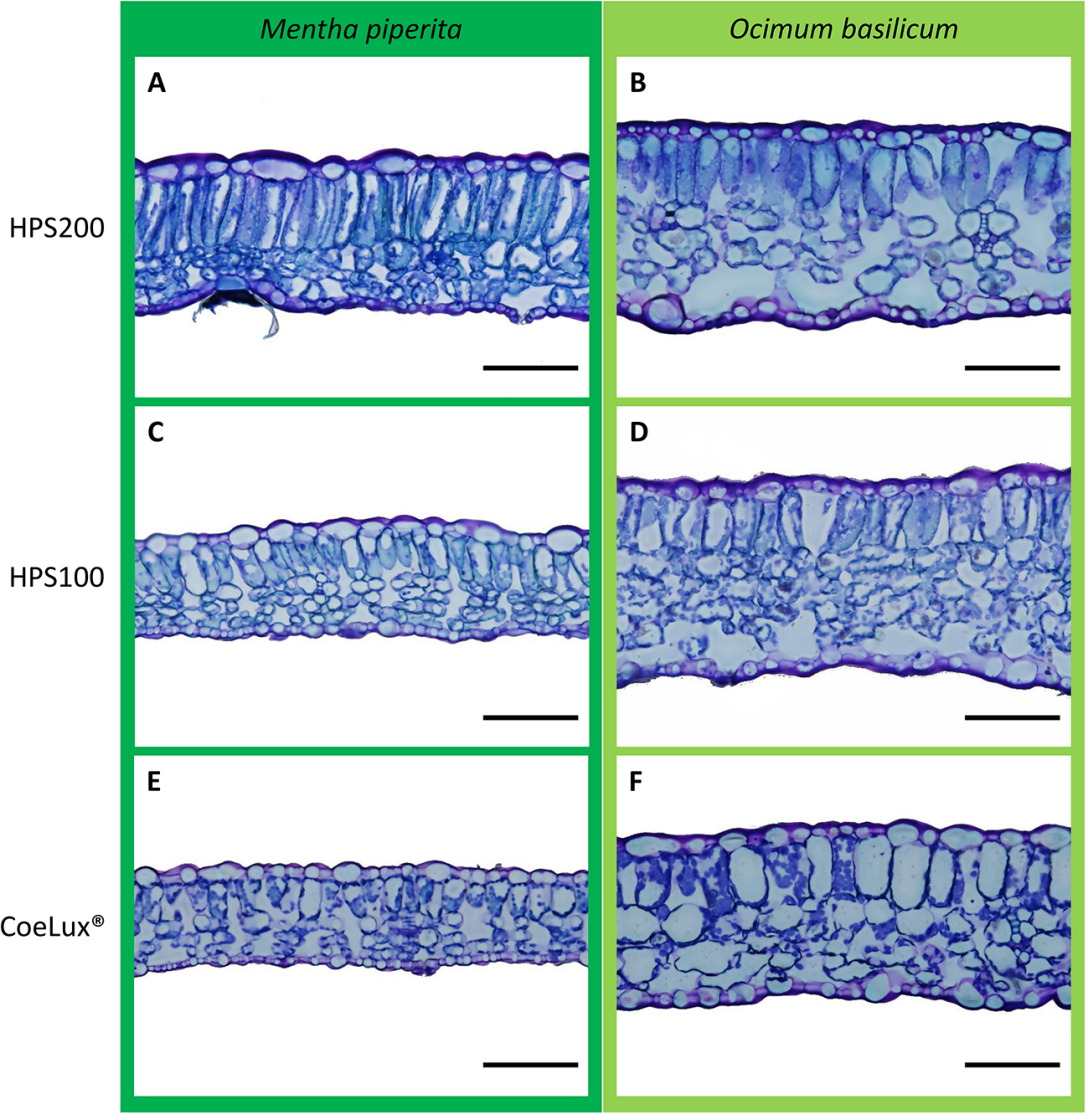
Comparison of representative leaves sections of *Mentha piperita* (dark green) and *Ocimum basilicum* (light green) grown under different light treatments, respectively **(A, B)** HPS200, **(C, D)** HPS100, and **(E, F)** CoeLux^®^. Images were obtained by optical microscopy using 10 µm slices of the central part of the fully expanded younger leaf. The black bar represents a 100 µm reference.

For each plant, the central part of the fully expanded younger leaf was fixed and conserved at 4°C in formaldehyde-glutaraldehyde fixative (Karnovsky, 1965) for analysis at the microscope. To analyze the cross-section anatomy, leaves were dehydrated in a graded series of ethanol solutions and embedded in the Technovit^®^ 7100 resin (Kulzer GmbH, Germany). Samples were sliced with a Leica SM 2400 microtome (Leica Biosystems, Germany) at a thickness of 10 µm, colored with 0.5% toluidine blue and photographed using an Olympus BX63 optical microscope equipped with an Olympus DP72 camera (Olympus scientific solutions, Japan). Digital images (**Figure 3**) were analyzed with the embedded cellSens imaging software (Olympus) to measure the whole leaf thickness (WLT) and the palisade thickness (PT). The ratio between WLT and PT was calculated. Five observation fields were randomly selected for each of the six biological replicates for a total of 30 fields per treatment.

**Figure 4:**
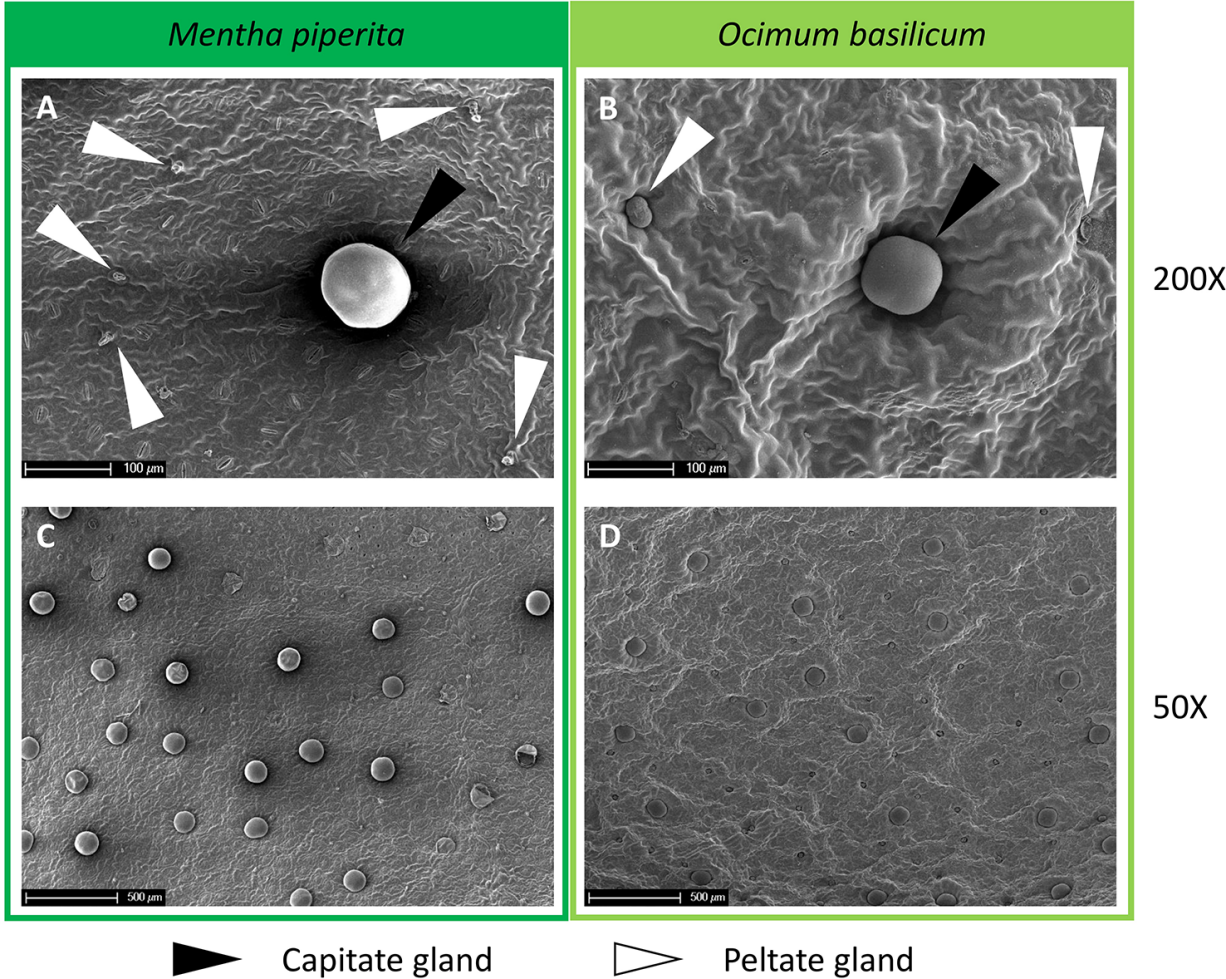
**(A, B)** SEM images at 200X magnification and **(C, D)** 50X magnification of the abaxial side of *Mentha piperita* (dark green) and *Ocimum basilicum* (light green) leaves. In panels **(A**, **B)**, capitate glands are marked with black arrows while peltate glands are marked with white arrows.

To count the number of trichomes on the surface of the leaves, the samples conserved in the fixative solution were washed twice with phosphate-buffered saline (PBS) and immersed in a solution 1:5 v/v composed of osmium tetroxide 4% and PBS for 1.5 h. Samples were subsequently washed twice with PBS, dehydrated in a graded series of ethanol solutions, and incubated in hexamethyldisilazane (two steps of 20 min each). Two samples for each leaf were processed and mounted on aluminum stubs to analyze both the abaxial and adaxial surfaces of the leaf. The Emitech K550 gold sputter coater was used to coat the samples with a 10 nm gold layer. Finally, the samples were observed with an XL30 FEG (Philips) scanning electron microscope (SEM) at 7 kV and images of five randomly selected observation fields per leaf side were acquired at 50X and 150X magnification to count both peltate and capitate glands (**Figure 4**). The total trichomes number, for the whole leaf area or per square centimeter, was calculated by summing the glands counted on the abaxial and adaxial surfaces of the leaf sample.

### Essential oil analysis

The fresh leaves of 10 different plants for each LT were pooled together and submitted to a 3 h hydrodistillation to extract the essential oils (EOs) according to the standard procedure described in the European Pharmacopoeia (Council of Europe et al., 2004). The EOs were dried over anhydrous sodium sulfate to remove traces of water and then stored in dark vials at 4°C before gas chromatography-mass spectrometry (GC-MS) analysis. The EOs yield was calculated and expressed as percentage on the dry weight of the extracted leaves. The characterization of all essential oils samples was determined with a gas chromatography system GC 86.10 Expander (Dani) equipped with an FID detector, an Rtx^®^-5 Restek capillary column (30 m × 0.25 mm i.d., 0.25 μm film thickness) (diphenyl-dimethyl polysiloxane), a split/splitless injector heated to 250°C and a flame ionization detector (FID) heated to 280°C. The column temperature was maintained at 40°C for 5 min, then programmed to increase to 250°C at a rate of 3°C/min and held, using an isothermal process, for 10 min; the carrier gas was He (1.0 mL/min); 1μL of each sample was dissolved in *n*-hexane (1:250 *n*-hexane solution) and injected. The experiment was repeated three times. The GC-MS analyses were performed on a Trace GC Ultra (Thermo Fisher Scientific) gas chromatography instrument equipped with an Rtx^®^-5 Restek capillary column (30 m × 0.25 mm i.d., 0.25 µm film thickness) and coupled with an ion-trap (IT) mass spectrometry (MS) detector Polaris Q (Thermo Fisher Scientific, Waltham, MA, USA). A Programmed Temperature Vaporizer (PTV) injector and a PC with a chromatography station Xcalibur (Thermo Fisher Scientific) were used. The ionization voltage was 70 eV; the source temperature was 250°C; full scan acquisition in positive chemical ionization was from m/z 40 up to 400 a.m.u. at 0.43 scan s^−1^. The GC conditions were the same as those described above for the gas chromatography (GC-FID) analysis. The identification of the essential oil components was based on the comparison of their Kovats retention indices (RIs) and LRI (linear retention indices) (Kovats, 1965), determined in relation to the t_R_ values of a homologous series of *n*-alkanes (C8–C20) injected under the same operating conditions as those described in the literature (Doll and Kratz, 1963). The MS fragmentation patterns of a single compound were those from the NIST 02, Adams and Wiley 275 mass spectral libraries (NIST/EPA/NIH, 2005) (McLafferty, 1998). The relative contents (%) of the sample components were computed as the average of the GC peak areas obtained in triplicate without any corrections (Grob and Barry, 2004).

### Statistical analysis

The statistical analysis was carried out with SPSS Statistics 25.0 (IBM) using the *post hoc* Dunnett’s test for multiple comparisons. In box plots, statistically significant differences (p< 0.05) between the means were marked with the letters a, b, and c. To characterize the levels of diversity in the chemical composition of the essential oils extracted from both *M. piperita* and *O. basilicum* (data in **Supplementary Tables 1, 2** respectively), we considered the classic Shannon entropy (*H*) (Pielou, 1975), with *p_i_* the proportion of the *i-th* of *k* compounds detected in the sample; and its relative version (*J*), the Pielou index (Mahmoud, 2021). The Shannon entropy can be used to compare the EOs diversity between treatments among the same plant species, while the Pielou index allows for a comparison of the diversity also between the two different plant species.


$$H=-\sum_{i=1}^{k}p_{i}\log\left(p_{i}\right)\;J=\frac{H}{log\left(k\right)}$$

Furthermore, to compare the compositions of different samples and to assess the levels of dissimilarities between light treatments, we calculated the *percent model affinity* (PMA) index as reported in (Ärje et al., 2016), where A and B denote two generic samples.


$$PMA=1-0.5\sum_{i=1}^{k}\left|p_{Ai}-p_{Bi}\right|$$

## Results

### Root and shoot morphological traits

**Figure 5:**
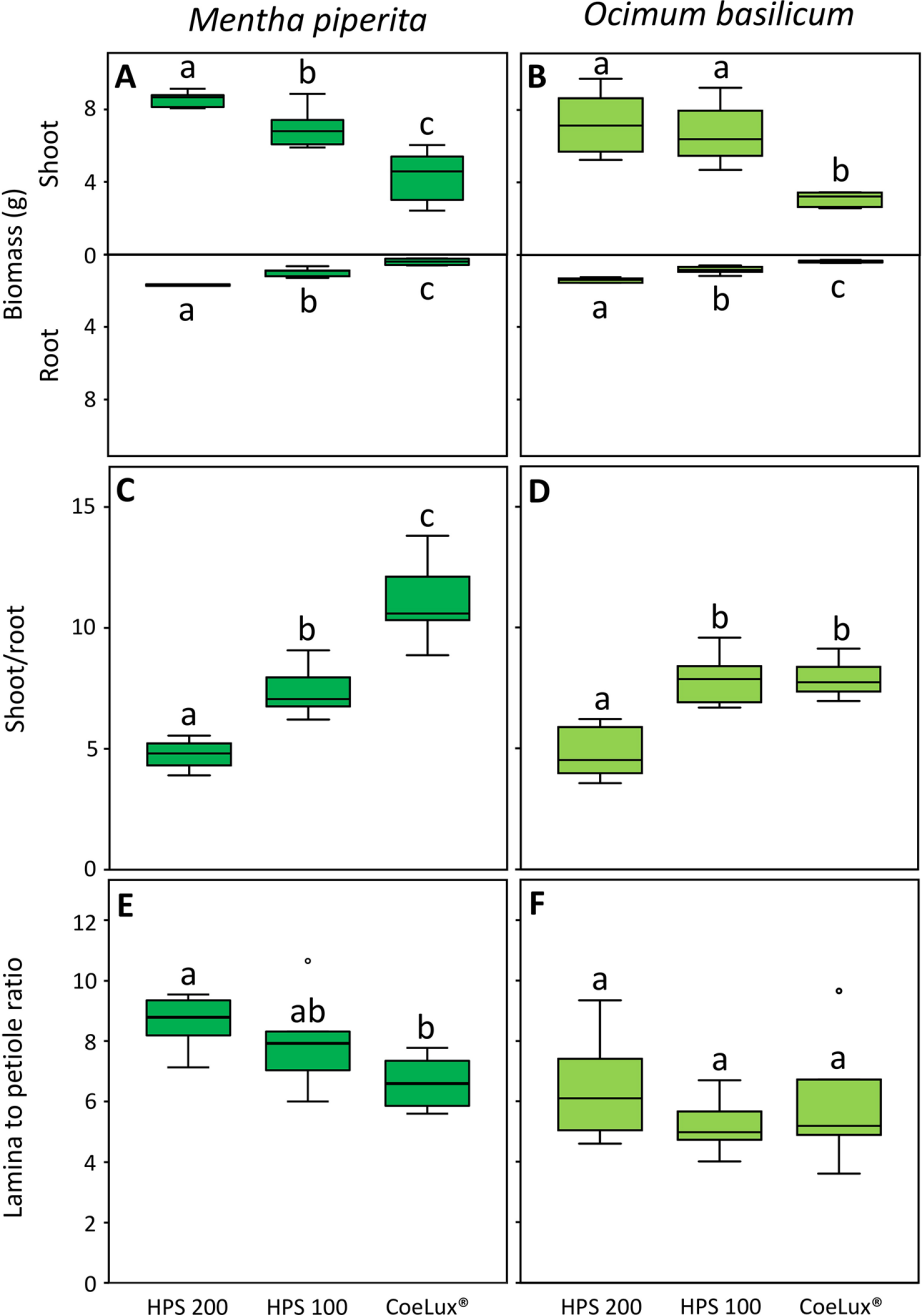
**(A, B)** shoot and root biomass (g), **(C, D)** shoot-to-root ratio, and **(E, F)** lamina-to-petiole length ratio measured under different light treatments for *Mentha piperita* (dark green) and *Ocimum basilicum* (light green) plants. Box plots represent n=6 biological repeats while letters represent statistically significant differences (p< 0.05). Vertical boxes represent approximately 50% of the observations and lines extending from each box are the upper and lower 25% of the distribution. Within each box, the solid horizontal line is the median value. Circles and asterisks represent respectively outliers and extreme outliers.

In *Mentha piperita* the biomass of both shoot (SB) and root (RB) was respectively the highest and the lowest in plants grown under the HPS200 and CoeLux^®^ LTs, while plants grown under the HPS100 LT had intermediate values (**Figure 5A**). The shoot-to-root ratio (S/R) was respectively the highest and the lowest in plants grown under the CoeLux^®^ and HPS200 LTs, while plants grown under the HPS100 LT had intermediate values (**Figure 5C**). The lamina-to-petiole length ratio (L/P) was respectively the highest and the lowest in plants grown under the HPS200 and CoeLux^®^ LTs, while plants grown under HPS100 had an intermediate median, but no statistically significant differences were observed with the other LTs (**Figure 5E**).

A similar pattern was observed in *Ocimum basilicum*. However, no statistically significant differences in the SB were observed between plants grown under the HPS100 and HPS200 LTs (**Figure 5B**) and in the S/R between plants grown under the HPS100 and CoeLux^®^ LTs (**Figure 5D**). The L/P showed no statistically significant differences between all light treatments tested (**Figure 5F**).

**Figure 6:**
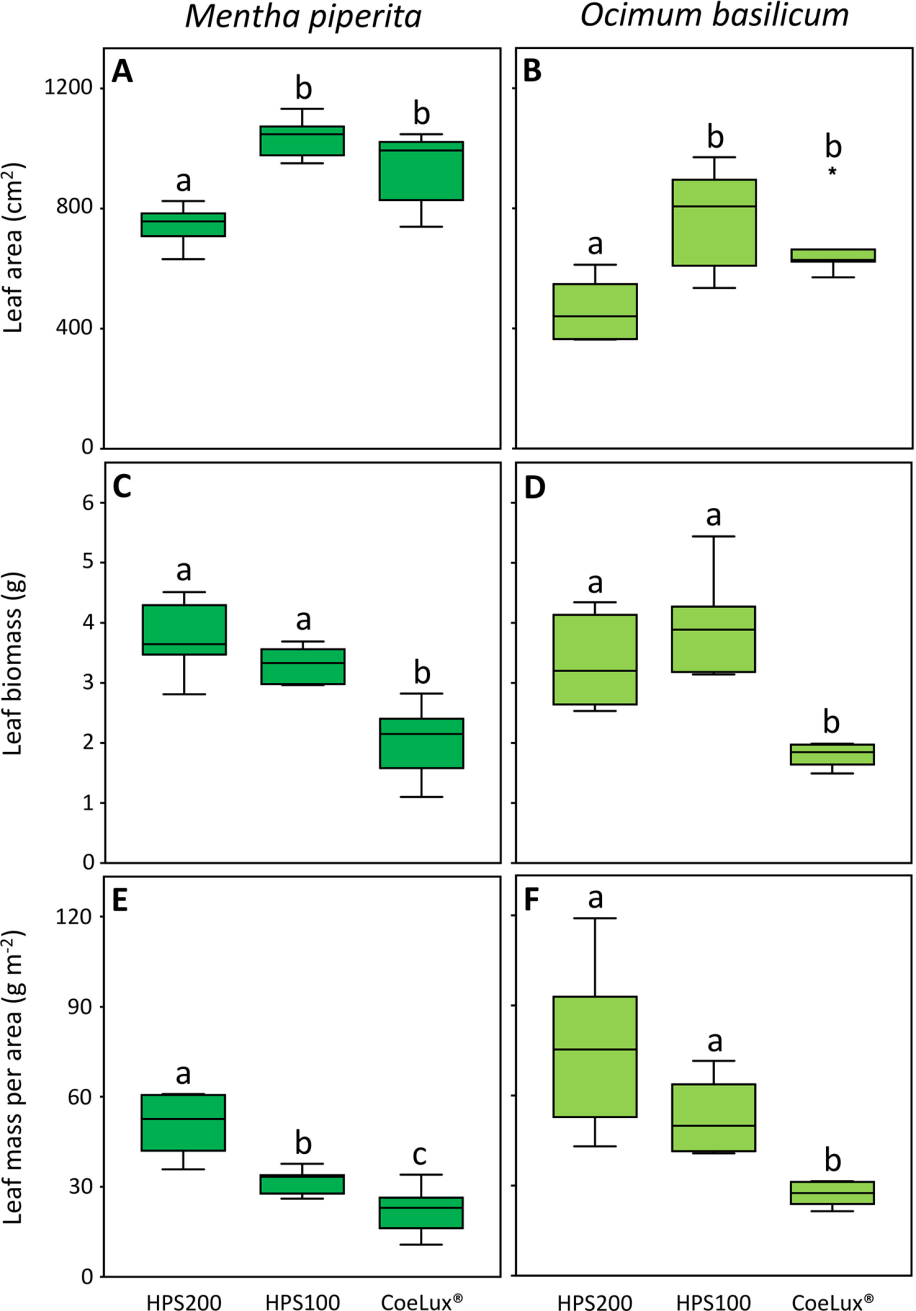
**(A, B)** leaf area (cm^2^), **(C, D)** leaf biomass (g), and **(E, F)** leaf mass per area (g m^-2^) measured under different light treatments for *Mentha piperita* (dark green) and *Ocimum basilicum* (light green) plants. Box plots represent n=6 biological repeats while letters represent statistically significant differences (p< 0.05). Vertical boxes represent approximately 50% of the observations and lines extending from each box are the upper and lower 25% of the distribution. Within each box, the solid horizontal line is the median value. Circles and asterisks represent respectively outliers and extreme outliers.

In *Mentha piperita* the leaf area (LA) was the highest in plants grown under the HPS100 and CoeLux^®^ LTs and the lowest in plants grown under the HPS200 LT (**Figure 6A**). The leaf biomass (LB) was the highest in plants grown under the HPS200 and HPS100 LTs and the lowest in plants grown under the CoeLux^®^ LT (**Figure 6C**). The leaf mass per area (LMA) was respectively the highest and the lowest in plants grown under the HPS200 and CoeLux^®^ LTs, while plants grown under the HPS100 LT had intermediate values (**Figure 6E**).

A similar pattern was observed in *Ocimum basilicum* for the LA and the LB (**Figures 6B, D**), while the LMA was the highest in plants grown under the HPS200 and HPS100 LTs and the lowest in plants grown under the CoeLux^®^ LT (**Figure 6F**).

### Leaf anatomical traits

**Figure 7:**
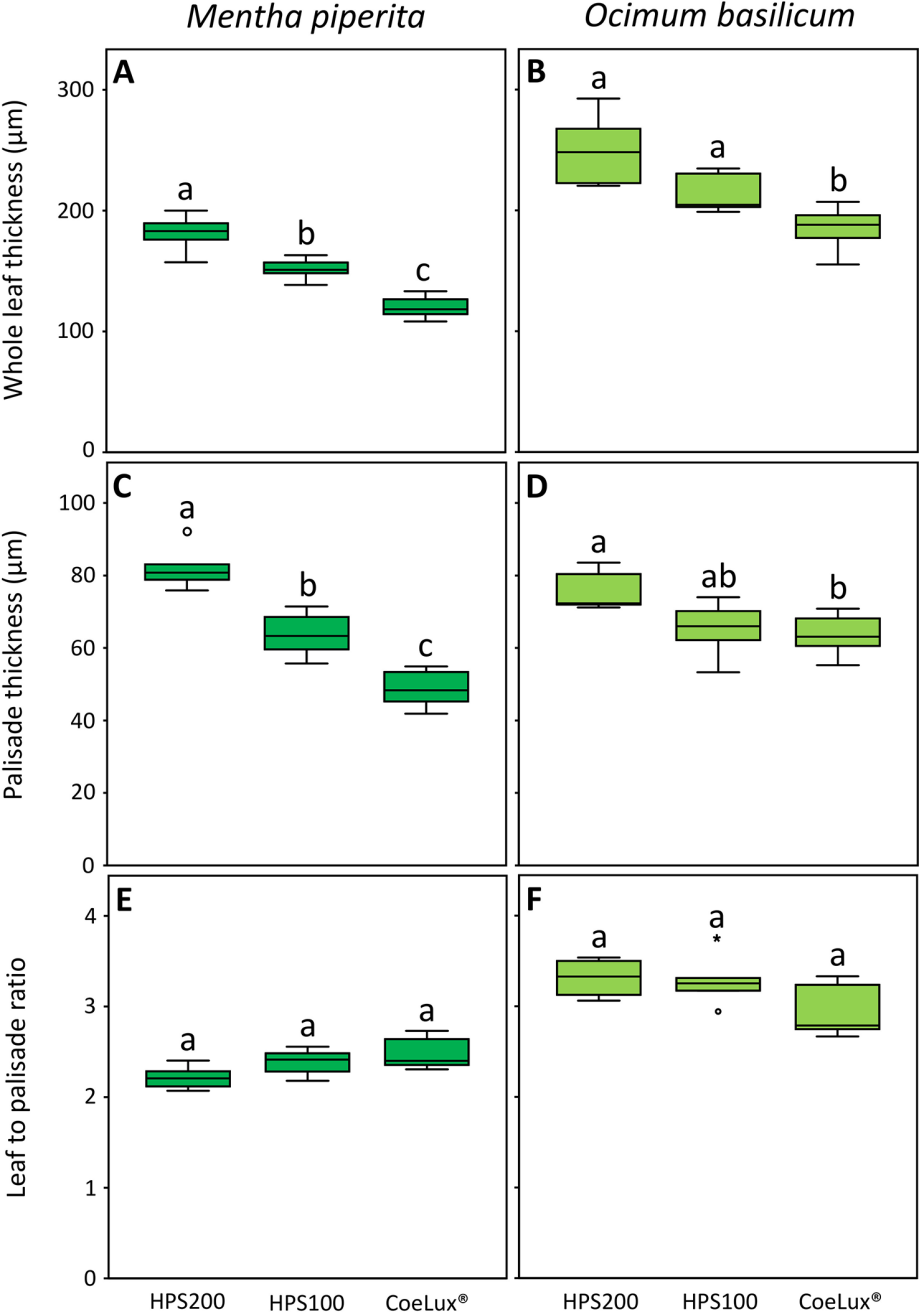
**(A, B)** whole leaf thickness (µm), **(C, D)** palisade thickness (µm), and **(E, F)** leaf-to-palisade ratio measured under different light treatments for *Mentha piperita* (dark green) and *Ocimum basilicum* (light green) plants. Box plots represent n=6 biological repeats while letters represent statistically significant differences (p< 0.05). Vertical boxes represent approximately 50% of the observations and lines extending from each box are the upper and lower 25% of the distribution. Within each box, the solid horizontal line is the median value. Circles and asterisks represent respectively outliers and extreme outliers.

In *Mentha piperita* the whole leaf thickness (WLT) was respectively the highest and the lowest in plants grown under the HPS200 and CoeLux^®^ LTs, while plants grown under the HPS100 LT had intermediate values (**Figure 7A**). The palisade thickness (PT) was respectively the highest and the lowest in plants grown under the HPS200 and CoeLux^®^ LTs, while plants grown under the HPS100 LT had intermediate values (**Figure 7C**). The ratio between WLT and PT showed no statistically significant differences in all light treatments tested (**Figure 7E**).

In *Ocimum basilicum* the WLT was the highest in plants grown under the HPS200 and HPS100 LTs and the lowest in plants grown under the CoeLux^®^ LT (**Figure 7D**). The PT was respectively the highest and the lowest in plants grown under the HPS200 and CoeLux^®^ LTs, while plants grown under HPS100 had an intermediate median, but no statistically significant differences were observed with the other LTs (**Figure 7D**). The ratio between WLT and PT showed no statistically significant differences in all light treatments tested (**Figure 7F**).

In both plant species, the number of peltate and capitate glands measured for the whole leaf area or calculated per square centimeter of leaf surface did not show differences in all light treatments tested (**Supplementary Figures 1** and **2**).

### Essential oil composition

*Mentha piperita* leaves showed a higher essential oils (EOs) yield (0.67-1.34%) when compared to *Ocimum basilicum* leaves (0.17-0.40%). In *M. piperita* the highest and lowest yields were reported in the SUN and HPS200 LTs respectively, while in *O. basilicum* the highest and lowest yields were reported in the HPS200 and SUN LTs (**Table 1**). A whole of 57 different molecular compounds was identified in the EOs of *M. piperita* leaves grown under different LTs (**Supplementary Table 1**). The highest number of compounds was observed in plants grown under the SUN LTs, while the lowest number of compounds was observed under the HPS200 LT. The CoeLux^®^ and HPS100 LTs had an intermediate number of molecules. Among the different LTs, the GC-MS TIC chromatogram coverage area ranged from 96.8% to 99.3% (**Table 1**). In *O. basilicum* a whole of 47 different molecular compounds was identified (**Supplementary Table 2**). The highest number of compounds was observed in plants grown under the SUN LTs, while the lowest number of compounds was observed under the CoeLux^®^ LT. The HPS200 and HPS100 LTs had an intermediate number of molecules. Among the different LTs, the GC-MS TIC chromatogram coverage area ranged from 96.8% to 99.1% (**Table 1**).

**Table 1:** Essential oils yield, expressed as percentage on the dry biomass of the leaves, number of compounds identified, and total area covered by the GC-MS TIC chromatograms for each essential oils sample extracted from plants subjected to different light treatments.

	*Mentha piperita*			*Ocimum basilicum*		
Light treatment	Yield (%)	No. of compounds	Total area	Yield (%)	No. of compounds	Total area
SUN	1.34	51	99.1%	0.17	47	98.6%
HPS200	0.67	45	99.2%	0.40	45	99.1%
HPS100	1.12	48	99.3%	0.24	46	96.8%
CoeLux^®^	0.94	47	96.8%	0.25	43	98.7%

**Figure 8:**
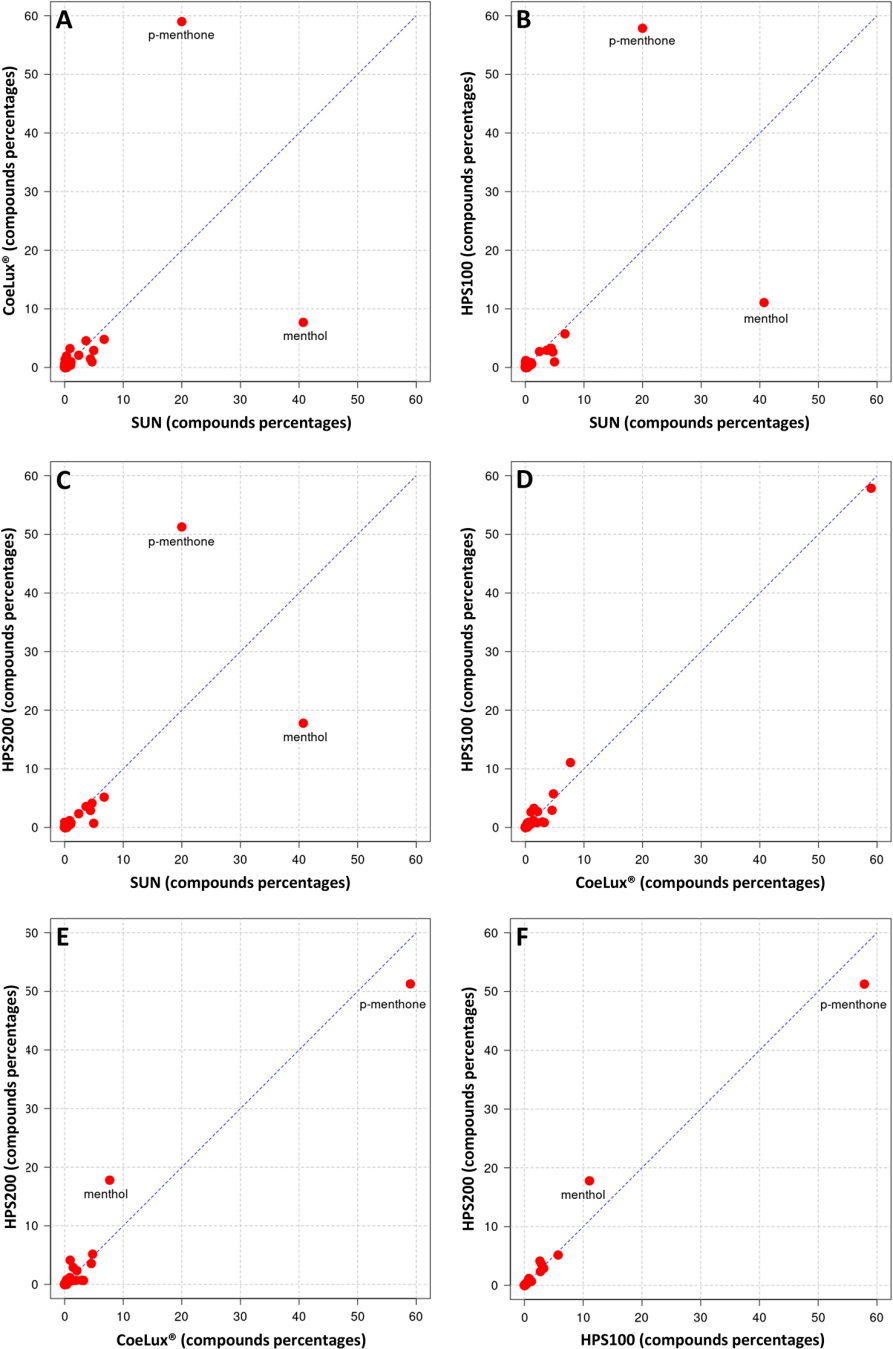
Cross-plot comparison of the biochemical composition in the essential oil of M. piperita plants grown under different light treatments. Respectively, **(A)** CoeLux® vs SUN, **(B)** HPS100 vs SUN, **(C)** HPS200 vs SUN, **(D)** HPS100 vs CoeLux®, **(E)** HPS200 vs CoeLux®, and **(F)** HPS200 vs HPS100. Points away from the bisector represent molecules differentially expressed in the two light treatments under analysis, while points on the bisector represent molecules present in the same percentage in both samples.

In *M. piperita* the EOs of plants grown under all three LTs under study (i.e., the CoeLux^®^, HPS200 and HPS100 LTs) were richer in *p*-menthone and poorer in menthol with respect to the SUN LT (**Figures 8A-C**). The EO of plants grown under the HPS100 LT showed a chemical composition comparable to that of CoeLux^®^ plants (**Figure 8D**), while plants grown under the HPS200 LT showed to have more menthol and less *p*-menthone with respect to the CoeLux^®^ and HPS100 LTs (**Figures 8E, F**). Furthermore, *cis-beta-*Guaiene and Eptadecane are produced almost exclusively under the CoeLux^®^ LT; and Pulegone, Terpinene-4-ol, *α*-Terpineol, Himachelen-ol, Cadinol-epi-*α*, and Cedren-13-ol<8> are produced only under artificial lighting (**Supplementary Table 1**). In contrast, 3-Octanol, *trans* Mentha-2,8-dien-1-ol, *β*-bourbonene, *α*-Muurolene, Guaiene<*trans-b->*, Selina-3,11-dien-6-a-ol, Himachalol, Eudesm-7(11)-en-4-ol are produced only under the SUN LT (**Supplementary Table 1**).

**Figure 9:**
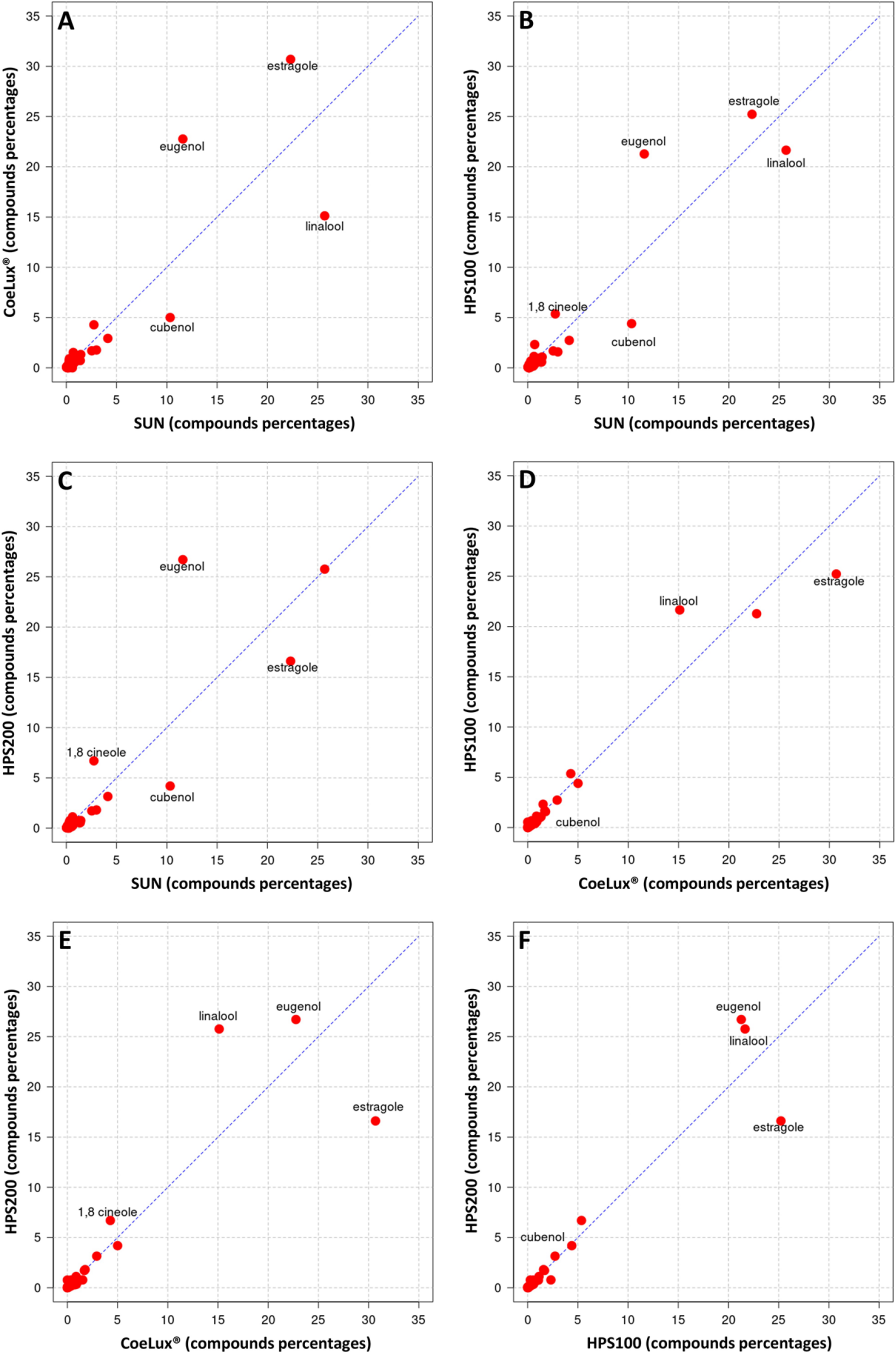
Cross-plot comparison of the biochemical composition in the essential oil of O. basilicum plants grown under different light treatments. Respectively, **(A)** CoeLux® vs SUN, **(B)** HPS100 vs SUN, **(C)** HPS200 vs SUN, **(D)** HPS100 vs CoeLux®, **(E)** HPS200 vs CoeLux®, and **(F)** HPS200 vs HPS100. Points away from the bisector represent molecules differentially expressed in the two light treatments under analysis, while points on the bisector represent molecules present in the same percentage in both samples.

In *O. basilicum* the EOs of plants grown under all three LTs under study (i.e., the CoeLux^®^, HPS200 and HPS100 LTs) were richer in Eugenol and poorer in Cubenol with respect to the SUN LT (**Figures 9A-C**). The EO of plants grown under the CoeLux^®^ LT were richer in Estragole, Borneol and Terpinen-4-ol, and poorer in Linalool with respect to the other LTs (**Figures 9A, D, E**). The EOs of plants grown under the HPS LTs showed to have more 1,8-cineole with respect to the CoeLux^®^ and SUN LTs (**Figures 9B-E**). Furthermore, plants grown under the CoeLux^®^ LT produced no α-Terpineol, which was reported for the other LTs (**Supplementary Table 2**).

Both in *M. piperita* and *O. basilicum*, the EOs composition assessed in plants grown under the CoeLux^®^ LT showed respectively the highest and lowest similarity with plants grown under the HPS100 LT and the SUN LT (**Table 2**). The SUN LT showed the highest and lowest similarity in both species with the HPS200 and the CoeLux^®^ LTs, respectively (**Table 2**). For *M. piperita* the highest similarity index value (0.899) was reported between the HPS200 and HPS100 LTs; for O. basilicum the highest similarity index value (0.893) was found between the CoeLux^®^ and HPS100 LTs.

**Table 2:** Similarity and diversity in biochemical compounds composition of *M. piperita* and *O. basilicum* essential oils were assessed with the PMA similarity index (minimum similarity =0; max similarity =1) and whit Shannon entropy and Pielou index (minimum diversity =0; max diversity =1). The standard error of three technical replicates is reported.

		Similarity				Diversity	
Plant species	Light Treatment	SUN	HPS200	HPS100	CoeLux^®^	Shannon entropy	Pielou index
*Mentha piperita*	SUN	1	0.649 ± 0.001	0.575 ± 0.002	0.521 ± 0.002	2.176 ± 0.007	0.536 ± 0.002
	HPS200		1	0.899 ± 0.001	0.822 ± 0.002	1.929 ± 0.005	0.475 ± 0.001
	HPS100			1	0.890 ± 0.002	1.865 ± 0.010	0.459 ± 0.002
	CoeLux^®^				1	1.887 ± 0.009	0.465 ± 0.002
*Ocimum basilicum*	SUN	1	0.784 ± 0.001	0.803 ± 0.001	0.757 ± 0.002	2.492 ± 0.019	0.644 ± 0.005
	HPS200		1	0.874 ± 0.001	0.802 ± 0.001	2.282 ± 0.003	0.589 ± 0.001
	HPS100			1	0.893 ± 0.001	2.354 ± 0.001	0.608 ± 0.000
	CoeLux^®^				1	2.317 ± 0.008	0.599 ± 0.002

In general, the *O. basilicum* EOs compositions had a higher level of diversity (Pielou index ranging between 0.589 and 0.644) than those observed for *M. piperita* (ranging between 0.459 and 0.536) (**Table 2**). For both species, the EOs composition of plants grown under the SUN LT resulted in the highest entropy and diversity index values (**Table 2**). In *M. piperita* the lowest diversity values resulted in the HPS100 LT, while in *O. basilicum* the lowest diversity values resulted in the HPS200 LT (**Table 2**).

## Discussion

### Morphological and anatomical traits

Previous studies have described the impact on the model plant *Arabidopsis thaliana* of the LED-sourced biophilic lighting systems CoeLux^®^ (Beatrice et al., 2021), characterized by low light intensity levels and low blue and high red light spectrum composition. In *A. thaliana*, the phenotype was characterized by low values of biomass, leaf area, and lamina-to-petiole ratio, symptomatic of the onset of an intense shade avoidance syndrome (SAS). In particular, plants grown at lower light intensities showed a delayed life cycle and were significantly smaller than plants grown with higher light intensities. Furthermore, within the same light intensity plants grown under the CoeLux^®^ light type showed an additional deficit when compared to control plants grown under the HPS light type (Beatrice et al., 2021). However, responses to such a peculiar light type might change among different plant species that differently modulate the response to light characteristics (Larsen et al., 2020). Therefore, we analyzed the morphological traits of two common aromatic plant species, *Ocimum basilicum* and *Mentha x piperita*, aiming to understand to which extent the CoeLux^®^ light type may enable their growth and development in closed environments. In addition, through the application of GC-MS analysis, we investigated the essential oils (EOs) composition as a possible indicator of the activation of diverse metabolic pathways.

Multiple morphological changes were observed in response to the different light types and intensities, which underscored a slightly different response among the two plant species analyzed. *M. piperita* showed a general suppression of both above and belowground biomass due to the lowering of the control light intensity and to the difference in the light spectrum occurring between the control (HPS) and the CoeLux^®^ light type. In the case of *O. basilicum*, the same pattern was observed for the root biomass (RB) while the shoot biomass (SB) was significantly lower only when comparing plants grown under the two light types and independently of the light intensity. These findings are in contrast with what was found in *O. basilicum* by (Jensen et al., 2018a) and (Larsen et al., 2020), who found no differences in SB between LTs with different proportions of red and blue light. Our results suggest that a higher proportion of blue light (24% instead of 14%) and a lower proportion of red light (35% instead of 41%), as in the case of the HPS light type, could increase the SB. Another difference between the two species was highlighted by the shoot-to-root ratio (S/R) values. Indeed, in the case of *M. piperita* S/R values were higher with the lowering of the light intensity (within the HPS light type) and between the HPS and the CoeLux^®^ light type (within the same light intensity). Under unfavorable light conditions, the plants increased the allocation of biomass to the shoots in an attempt to enhance the uptake of the most limiting factor, light (Poorter et al., 2012). On the contrary, in the case of *O. basilicum*, the only difference was detected with the decrease in light intensity of the HPS light type, while no differences were observed among different light spectra originating from the HPS and the CoeLux^®^ light types. These findings highlight that the growth of *O. basilicum*, especially for the aboveground organs, is influenced to a lower extent by both light intensity and spectra.

The leaf area of both *M. piperita* and *O. basilicum* was higher with the lowering of the light intensity (within the HPS light type), while no difference was detected among the two different light spectra (within the same light intensity). Tabbert et al. (2022) attributed the greater leaf expansion observed in *M. piperita* to the low blue-to-green ratio (B/G) of their LTs, while Larsen et al. (2020) described a decrease in leaf area with the increase of the fraction of blue light in *O. basilicum*. However, we found no significant differences in the leaf area between the two light spectra of our LTs, despite having a higher B/G in the HPS light type (0.83) with respect to the CoeLux^®^ light type (0.50) due to a higher blue fraction composing the HPS light spectra (24%) in respect to the CoeLux^®^ light spectra (14%) and equal green fractions (24%). In both species, a lowering in the leaves’ biomass was detected in plants grown under the CoeLux^®^ light type when compared to plants grown under the HPS light type (within the same light intensity), but no differences were observed with the lowering of the light intensity. However, when the leaf mass per area (LMA) was calculated, further differences between the two plant species were detected. Indeed, in the case of *M. piperita* values were lower with the lowering of the light intensity (within the HPS light type) and between the HPS and the CoeLux^®^ light type (within the same light intensity), while in *O. basilicum* a lower value was observed only in the case of the CoeLux^®^ light type (characterized by a higher red and lower blue fraction). These data are in contrast with what was observed by Jensen et al. (2018), who found no differences in the LMA of *O. basilicum* grown under LTs with different fractions of red and blue light.

Further investigations showed that a lower whole leaf thickness (WLT) could be the reason for the lower LMA observed in both species (**Figure 6**). Indeed, the reduction in leaf thickness is a typical response observed during the SAS onset (Smith and Whitelam, 1997) for a plant dealing with a reduction in light intensity or a change in the light spectrum (e.g., lowering in the red/far-red ratio) (Franklin et al., 2005). In *M. piperita* WLT values were lower with the lowering of the light intensity (within the HPS light type) and between the HPS and the CoeLux^®^ light type (within the same light intensity). On the contrary, in *O. basilicum* a lower value was observed only in the case of the CoeLux^®^ light type, showing that a lowering in light intensity is not significantly affecting the leaf thickness in this species. When the leaf palisade was investigated, it was observed that for both species its structure remains intact also at the lowest light intensity (**Figure 3**), but the thickness (PT) reduced significantly with different magnitudes among the two plant species. Indeed, in the case of *M. piperita*, the PT values were lower with the lowering of the light intensity (within the HPS light type) and between the HPS and the CoeLux^®^ light type (within the same light intensity). On the contrary, in the case of *O. basilicum*, a significant PT reduction was detected only when the interplay between light intensity decrease and light spectrum change is considered (i.e., between the HPS200 and CoeLux^®^ LTs). These trends seem consistent with the WLT reduction, as no statistically significant differences were observed in the ratio between whole leaf thickness and palisade thickness. Apparently, all leaf organs undergo an even thickness reduction in response to the biophilic light conditions tested.

In *A. thaliana* the lamina-to-petiole length ratio (L/P) reduction is considered a hallmark response to unfavorable light (Keller et al., 2011). A low lamina-to-petiole ratio and a high shoot-to-root ratio are characteristic responses for improving light collection under unfavorable light conditions (Poorter et al., 2012). Indeed, under low light conditions, *A. thaliana* develops a longer petiole and a shorter lamina in an effort to better align the leaves toward the light source and collect more light for photosynthetic activity (Beatrice et al., 2021). However, in *M. piperita* an L/P reduction was detected only with the interplay between light intensity decrease and light spectra change (i.e., between the HPS200 and CoeLux^®^ LTs), while no differences were observed within plants grown under the HPS light type with a reduction in light intensity or within plants grown at the same light intensity but with a different spectrum. Furthermore, *O. basilicum* showed no statistically significant differences under all LTs tested, demonstrating that the responses to biophilic lighting of this plant species are more focused on strategies like leaf area expansion, instead of leaf movement toward better light conditions.

### Essential oils composition

#### Mentha piperita

Previous studies found that the age of the leaves and their state of maturity are the principal causes of essential oil composition variance among *M. piperita* plants (Lawrence, 2007). Hefendehl (1962) found that as the leaves increased in age, the reduction of menthone in menthol increased proportionally as the result of direct conversion as the biosynthetic pathway proceeded (Kokkini, 1991). Furthermore, younger leaves were found to have higher levels of pulegone, a menthone precursor (Tabbert et al., 2022). Under our LTs we observed a higher concentration of menthone at the lower light intensities irradiated by the CoeLux^®^ and HPS100 LTs, while the lower concentration was observed in plants grown under natural sunlight (SUN LT). The concentration of pulegone followed a similar pattern, while the concentration of menthol followed an inverse pattern, with the higher concentration observed under natural sunlight. Among the less abundant molecules, lower concentrations of neomenthol, 1,8-cineole and limonene were found in the oil extracted from plants grown under the CoeLux^®^ LT while higher concentrations were found in the control LTs and especially in the SUN LT. Von Schantz and Norri (1968) harvested plants at different development times-points and found lower concentrations of these molecules in oils extracted from juvenile plants, while higher concentrations were produced in flowering plants (Lawrence, 2007). On the other hand, the concentration of the commercially undesirable oil component menthofurane (Lawrence, 2007) showed no significant differences between the LTs analyzed. The detected percentages of menthofurane (0.37% to 0.53% depending on the LT) and pulegone (0.08% to 1.36%) were abundantly below the legal limits of 9% and 4% (Council of Europe et al., 2004), as both compounds have been shown to be hepatotoxic (Malekmohammad et al., 2021). These data suggest that despite the similar treatment time, plants grown under artificial lighting, and in particular plants grown under the CoeLux^®^ LT, had a higher proportion of immature leaves that shaped the composition of the main components of the plant’s essential oils. Among the plants grown under the HPS light type, plants grown under the higher light intensity (200 µmol m^-2^s^-1^) seem to have leaves with a more mature chemical profile than plants grown with lower light intensity (100 µmol m^-2^s^-1^). These findings are in line with those recently reported by Tabbert et al. (2022), who observed an incomplete biosynthesis of menthol under the low light intensities produced by LED light sources in indoor vertical farms. Under natural sunlight, plants are subjected to light conditions with pronounced changes during the day and during the whole growth period, both in terms of photoperiod, spectrum, and light intensity, in contrast with the constant conditions found under artificial lighting. In particular, the daily light integral is higher under natural sunlight and high light intensities are reached in the brighter hours of sunny days (> 2000 µmol m^-2^s^-1^), which may also result in photoinhibition of photosynthesis and destruction of the photosynthetic apparatus (Long and Humphries, 1994). Under these light conditions, *M. piperita* plants produced higher EOs yields with a higher proportion of menthol.

The similarity analysis (**Table 1**) confirmed that plants grown under our artificial lighting systems produced similar essential oils. In particular, the highest similarities were found between plants sharing the same light type (HPS200 and HPS100) or the same light intensity (HPS100 and CoeLux^®^). The lack of similarity between the SUN LT and the other three LTs is mainly due to the reduction of a consistent quantity of menthone in menthol that was observed only under natural sunlight.

In *M. piperita*, the desirable oil quality includes menthol levels above 50% and menthofurane and pulegone levels below 4% and 2%, respectively (Lawrence, 2007). In our case and in terms of commercial use, the essential oils extracted from plants grown under artificial illumination seem to have a lower value due to the lower concentration of menthol, the most commercially desirable molecule, as menthol is used in a variety of products including toothpaste, chewing gum, sweets, beverages, and drugs (Lawrence, 2007). However, the overall quality of the essential oil allows us to speculate that the CoeLux^®^ lighting system may represent a suitable alternative to conventional indoor lighting systems for the growth of *M. piperita* plants. Thus, we might assert that the CoeLux^®^ lighting system coupled with *M. piperita* plants may push forward the design of future biophilic environments that consider both plants and artificial sunlight.

#### Ocimum basilicum

The typical sweet basil aroma is the result of a complex combination of dozens of secondary metabolites (Pirmoradi et al., 2013), among these eugenol has a strong clove-related flavor, linalool has a sweet and floral aroma, while 1,8-cineole (eucalyptol) has a spicy and camphor-like aroma (Carvalho et al., 2016). The essential oils of *O. basilicum* are usually characterized by high concentrations of linalool and estragole (methyl chavicol), besides other characteristic oil compounds like 1,8-cineole, eugenol, and methyl eugenol (Hiltunen and Holm, 1999). In our study, plants grown under the SUN LT showed high concentrations of linalool and estragole and lower concentrations of the other compounds, while plants grown under the artificial LTs showed high concentrations not only of estragole and linalool but also of eugenol and 1,8-cineole. Furthermore, cubenol, *α-trans-*bergamotene, g-cadinene, and germacrene D were also found in relatively high abundance in our artificial LTs. However, it should be pointed out that different *O. basilicum* cultivars differ in the relative abundance of their characterizing compounds (Marotti et al., 1996). In experiments with shaded *O. basilicum* plants (i.e., lower light intensity), higher shading resulted in higher methyl eugenol production (Chang et al., 2008), a phenylpropanoid that is thought to be carcinogenic (Dörr et al., 2020). We also found higher concentrations of methyl eugenol in the two LTs with the lower light intensity (i.e., CoeLux^®^ and HPS100). On the other hand, in these two LTs, we found higher concentrations of camphor, a monoterpene that is thought to have anticancer activity (da Silva et al., 2022). A study on the medicinal plant *Lippa rotundifolia* showed that monochromatic blue light from LEDs increased the content of monoterpenes such as myrcene and limonene (de Hsie et al., 2019). In our experiment, we found the highest concentrations of myrcene and limonene under the HPS100 and HPS200 LTs, characterized by a higher blue component with respect to the CoeLux^®^ LT (**Figure 2**). Furthermore, Carvalho et al. (2016) observed that growing *O. basilicum* under monochromatic red light produced an enhanced production of phenylpropanoids (i.e., estragole and eugenol), this finding is in line with what was observed in our study, as a higher fraction of red light resulted in a higher concentration of estragole and eugenol under the CoeLux^®^ LT in respect to the control LTs.

Overall, the diversity of the *O. basilicum* LTs was higher than the diversity observed in the *M. piperita* LTs. This differential characteristic among the two plant species is due to the higher abundance of the main basil EOs components when compared with mint, which has essential oils characterized by the presence of one main chemical compound, respectively menthone or menthol. Furthermore, in both species, the highest diversity was observed under the SUN LT, probably due to the higher number of compounds produced under natural environmental conditions. As previously observed in *M. piperita*, the statistical analysis (**Table 1**) found the highest similarities between plants sharing the same light type (HPS200 and HPS100) or the same light intensity (HPS100 and CoeLux^®^). However, differently from *M. piperita*, higher similarities were observed between the SUN LT and the other three LTs. Thus, we may assert that in terms of *O. basilicum* essential oils quality, the CoeLux^®^ lighting systems could be a valid alternative to both conventional HPS lighting systems and natural sunlight, providing a powerful tool for improving the design of biophilic environments.

## Conclusions

This research suggested that both *M. piperita* and *O. basilicum* undergo a reduction in plant growth when irradiated by the CoeLux^®^ lighting systems. However, *O. basilicum* showed to perform better with lower light intensity and the CoeLux^®^ spectral composition. Nevertheless, both plant species were able to thrive under the CoeLux^®^ lighting systems, as no mortality or diseases were observed. The similarity analysis performed on the essential oils composition showed that plants grown under the CoeLux^®^ light type produced oils with high similarity to those produced under the control light type in both plant species, suggesting that the CoeLux^®^ lighting systems could be a valid alternative to conventional HPS lighting systems. Furthermore, in *O. basilicum* a high similarity was detected also between the CoeLux^®^ light treatment and the control plants grown under natural sunlight.

Overall, it can be speculated that the growth of the aromatic plants *M. piperita* and *O. basilicum* under the CoeLux^®^ lighting systems is a feasible strategy to improve biophilic approaches in closed environments that include both plants and artificial sunlight. Among the two plant species analyzed, *O. basilicum* showed an overall better performance in terms of both morphological traits and essential oil composition. Further studies could explore the effect of soil type, fertilizers, amendments, or photoperiod length to find technical solutions that could improve the growth of these aromatic plant species under the biophilic LED-sourced lighting systems CoeLux^®^ and boost the production of health and commercial valuable essential oils components. In particular, technical solutions able to enhance the light intensity irradiating plants could boost the production of biomass and enhance the quality of essential oils (e.g., increasing the menthol concentration in *M. piperita* EOs).

## Data availability statement

The original contributions presented in the study are included in the article/**Supplementary Material**. Further inquiries can be directed to the corresponding author.

## Author contributions

AM and DC: conceptualization. AM and DC: primary funding. AM: supervision and important insight. PB and AM: study plan development. PB, FF, GS: methodology, morphological and chemical analysis. MR and PB: SEM analysis. FD and PB: data analysis. PB, FD, GS, and AM: data interpretation. PB: writing the original draft. AM and PB: revision process and finalization. All authors contributed to the article and approved the submitted version.
