# Supplementary material for: Light spectra of biophilic LED-sourced system modify essential oils composition and plant morphology of Mentha piperita L. and Ocimum basilicum L
Source: Front Plant Sci. 2023 Jan 19;14:1093883. doi: 10.3389/fpls.2023.1093883 (PMC9893021; doi:10.3389/fpls.2023.1093883)
Supplement: Supplementary file 1 [file DataSheet_1.docx]

# Supplementary Material

**
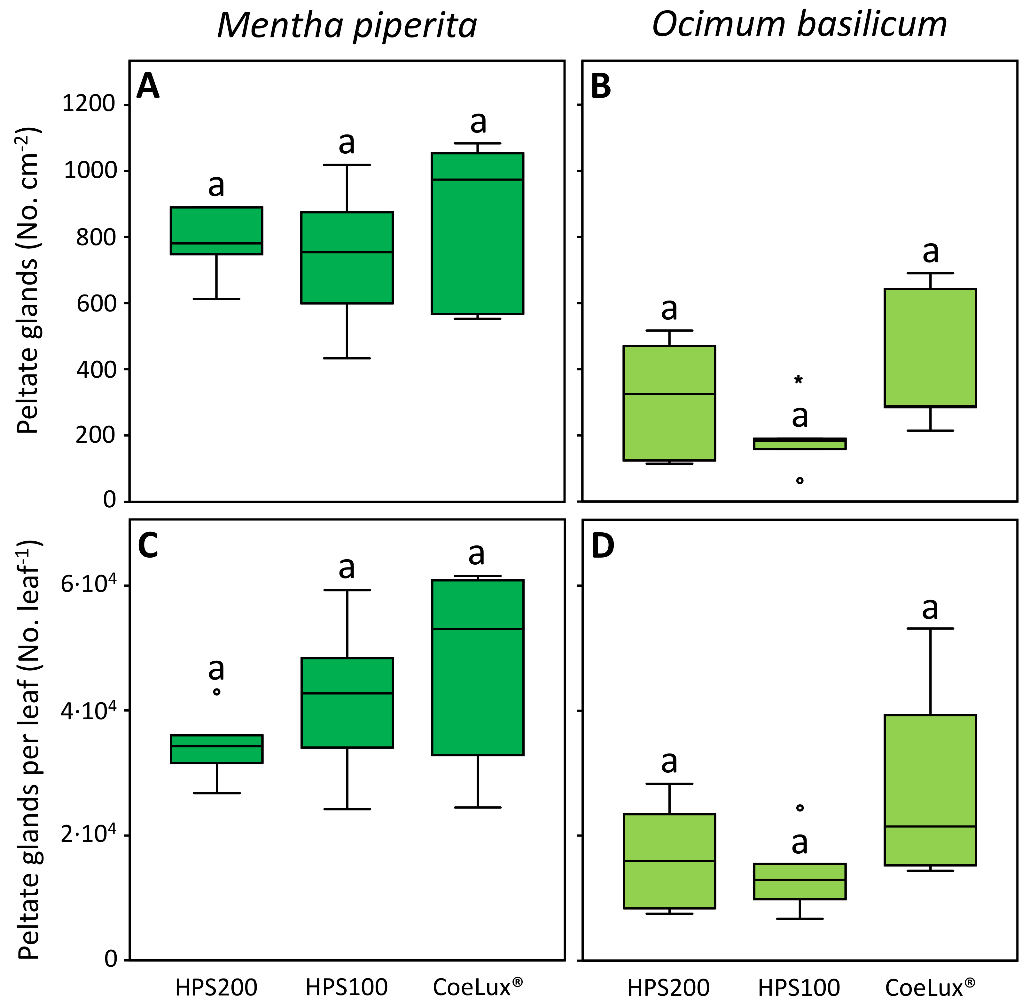
**

**Supplementary figure 1.** (**A**, **B**) peltate glands number per square centimeter and (**C**, **D**) peltate glands number per leaf measured under different light treatments for *Mentha piperita* (dark green) and *Ocimum basilicum* (light green) plants. Box plots represent n=6 biological repeats while letters represent statistically significant differences (p < 0.05). Vertical boxes represent approximately 50% of the observations and lines extending from each box are the upper and lower 25% of the distribution. Within each box, the solid horizontal line is the median value. Circles and asterisks represent respectively outliers and extreme outliers.

**
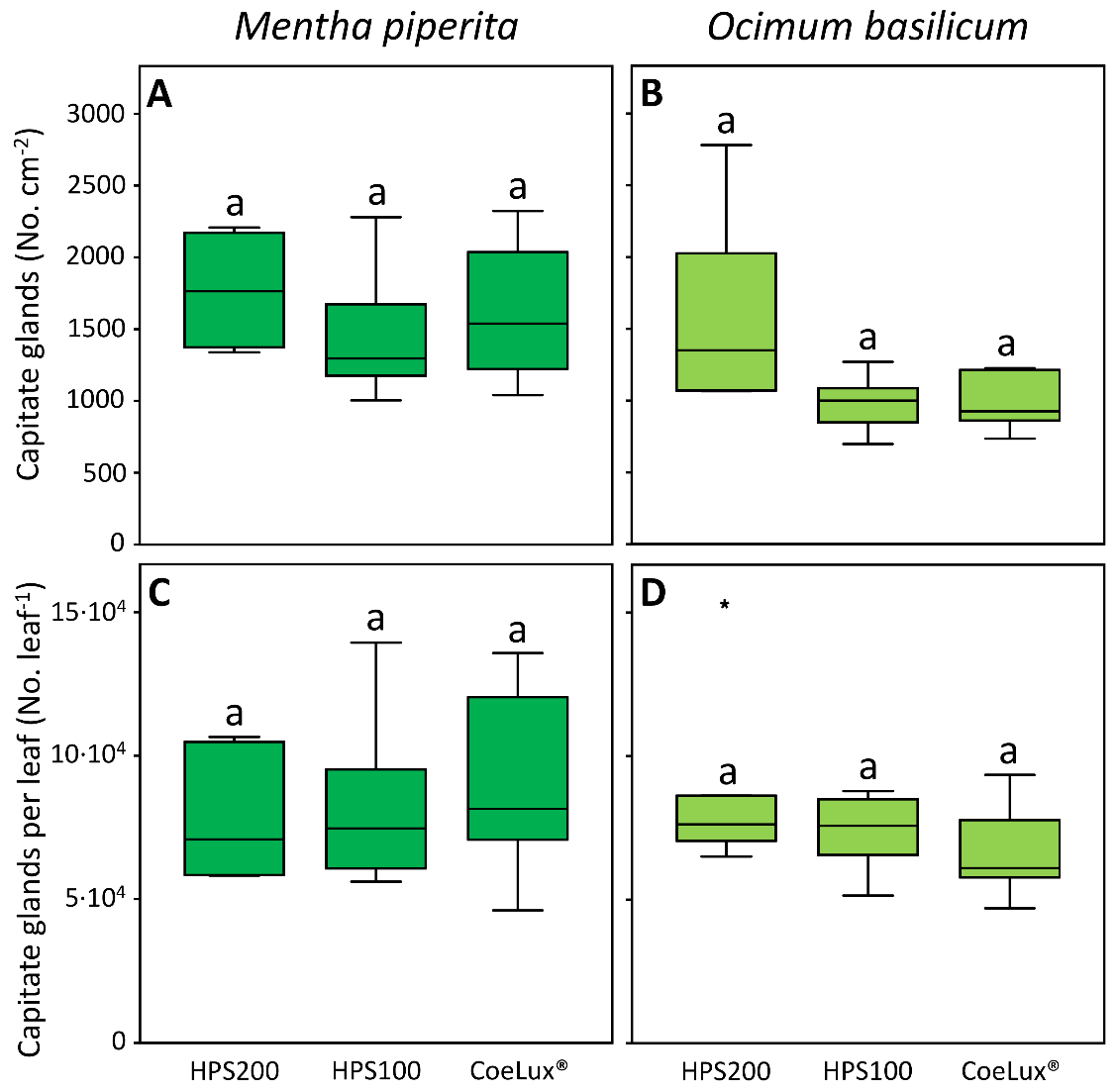
**

**Supplementary figure 2.** (**A**, **B**) capitate glands number per square centimeter and (**C**, **D**) capitate glands number per leaf measured under different light treatments for *Mentha piperita* (dark green) and *Ocimum basilicum* (light green) plants. Box plots represent n=6 biological repeats while letters represent statistically significant differences (p < 0.05). Vertical boxes represent approximately 50% of the observations and lines extending from each box are the upper and lower 25% of the distribution. Within each box, the solid horizontal line is the median value. Circles and asterisks represent respectively outliers and extreme outliers.

**Supplementary Table 1.** Chemical composition and chromatogram peak area of the essential oils extracted from *M. piperita* grown under different LTs. The compounds are listed according to their elution on a Rtx®-5 Restek capillary column.

| **No.** | **Compound** | **Exp. RI** | **Ref. RI** | **SUN**  **(area % ± SE)** | **HPS200**  **(area % ± SE)** | **HPS100**  **(area % ± SE)** | **CoeLux®**  **(area % ± SE)** | **Abbr.** |
| --- | --- | --- | --- | --- | --- | --- | --- | --- |
| 1 | *α*-Pinene | 936 | 939 | 0.44 ± 0.03 | 0.39 ± 0.02 | 0.42 ± 0.01 | 0.26 ± 0.02 | BM |
| 2 | Sabinene | 976 | 975 | 0.51 ± 0.03 | 0.32 ± 0.02 | 0.36 ± 0.02 | 0.20 ± 0.00 | BM |
| 3 | *β*-Pinene | 977 | 979 | 0.66 ± 0.03 | 0.56 ± 0.01 | 0.57 ± 0.02 | 0.33 ± 0.02 | BM |
| 4 | Myrcene | 993 | 990 | 0.27 ± 0.01 | 0.25 ± 0.01 | 0.29 ± 0.01 | 0.15 ± 0.01 | AM |
| 5 | 3-Octanol | 998 | 991 | 0.06 ± 0.01 | t | t | t | OT |
| 6 | *δ*-3-Carene | 1016 | 1011 | 0.13 ± 0.01 | 0.15 ± 0.06 | 0.23 ± 0.02 | 0.16 ± 0.02 | BM |
| 7 | *p*-Cymene | 1026 | 1024 | 0.15 ± 0.01 | t | t | 0.05 ± 0.00 | MM |
| 8 | Limonene | 1031 | 1029 | 4.68 ± 0.14 | 4.13 ± 0.04 | 2.64 ± 0.03 | 0.95 ± 0.01 | MM |
| 9 | 1-8-Cineole | 1034 | 1031 | 4.41 ± 0.18 | 2.89 ± 0.01 | 3.27 ± 0.21 | 1.46 ± 0.08 | BMO |
| 10 | *cis*-Ocimene | 1043 | 1037 | 0.38 ± 0.03 | 0.78 ± 0.60 | 0.74 ± 0.04 | 0.32 ± 0.01 | AM |
| 11 | *trans* Ocimene | 1053 | 1050 | 0.11 ± 0.01 | 0.23 ± 0.01 | 0.22 ± 0.00 | 0.12 ± 0.01 | AM |
| 12 | γ-Terpinene | 1062 | 1059 | 0.22 ± 0.00 | 0.36 ± 0.01 | 0.44 ± 0.01 | 0.63 ± 0.00 | MM |
| 13 | *p*-Mentha-3,8-diene | 1071 | 1072 | 1.02 ± 0.02 | 0.55 ± 0.01 | 0.73 ± 0.01 | 0.38 ± 0.01 | MM |
| 14 | *p*-Mentha-2,4(8)-diene | 1089 | 1088 | 0.10 ± 0.00 | 0.13 ± 0.00 | 0.14 ± 0.00 | 0.09 ± 0.00 | MM |
| 15 | Linalool | 1104 | 1096 | 0.33 ± 0.00 | 0.69 ± 0.01 | 0.80 ± 0.01 | 1.96 ± 0.02 | AMO |
| 16 | Allo-Ocimene | 1133 | 1132 | 0.09 ± 0.03 | 0.25 ± 0.60 | 0.24 ± 0.05 | 0.08 ± 0.02 | AM |
| 17 | *cis* Mentha-2,8-dien-1-ol | 1143 | 1137 | 0.06 ± 0.01 | 0.05 ± 0.00 | 0.12 ± 0.01 | 0.02 ± 0.01 | MMO |
| 18 | *trans* Mentha-2,8-dien-1-ol | 1145 | 1140 | 0.04 ± 0.01 | t | t | t | MMO |
| 19 | Camphor | 1149 | 1146 | 0.05 ± 0.01 | 0.12 ± 0.01 | 0.09 ± 0.02 | 0.20 ± 0.01 | BMO |
| 20 | *p*-Menthone | 1159 | 1152 | 19.99 ± 0.09 | 51.26 ± 0.18 | 57.86 ± 0.31 | 59.00 ± 0.34 | MMO |
| 21 | Menthofurane | 1166 | 1164 | 0.42 ± 0.04 | 0.37 ± 0.03 | 0.39 ± 0.03 | 0.53 ± 0.06 | BMO |
| 22 | Neomenthol | 1169 | 1165 | 6.75 ± 0.04 | 5.17 ± 0.13 | 5.73 ± 0.07 | 4.78 ± 0.31 | MMO |
| 23 | Menthol | 1178 | 1171 | 40.72 ± 0.39 | 17.78 ± 0.6 | 11.07 ± 0.05 | 7.69 ± 0.07 | MMO |
| 24 | Terpinen-4-ol | 1180 | 1177 | t | 0.88 ± 0.01 | 0.90 ± 0.05 | 0.61 ± 0.07 | BMO |
| 25 | *α*-Terpineol | 1194 | 1188 | t | 0.28 ± 0.01 | 0.23 ± 0.01 | 0.12 ± 0.01 | MMO |
| 26 | Myrtenal | 1198 | 1195 | 0.03 ± 0.01 | t | 0.10 ± 0.01 | t | BMO |
| 27 | Pulegone | 1244 | 1237 | 0.08 ± 0.00 | 0.67 ± 0.01 | 1.19 ± 0.01 | 1.36 ± 0.01 | MMO |
| 28 | Piperitone | 1260 | 1252 | 2.41 ± 0.03 | 2.35 ± 0.01 | 2.71 ± 0.02 | 2.08 ± 0.03 | MMO |
| 29 | Menthyl acetate <*neo*> | 1279 | 1273 | 0.32 ± 0.01 | t | 0.06 ± 0.01 | t | MMO |
| 30 | Menthyl acetate | 1296 | 1295 | 4.97 ± 0.00 | 0.71 ± 0.00 | 0.95 ± 0.01 | 2.90 ± 0.01 | MMO |
| 31 | *δ*-Elemene | 1339 | 1338 | 0.29 ± 0.10 | 0.2 ± 0.07 | 0.21 ± 0.04 | 0.35 ± 0.60 | MS |
| 32 | Copaene | 1377 | 1376 | 0.06 ± 0.01 | 0.04 ± 0.00 | 0.04 ± 0.00 | 0.05 ± 0.01 | BS |
| 33 | *β*-Bourbonene | 1386 | 1388 | 0.36 ± 0.00 | t | t | t | BS |
| 34 | *β*-Elemene | 1393 | 1390 | 0.17 ± 0.04 | 0.12 ± 0.01 | 0.13 ± 0.01 | 0.16 ± 0.02 | MS |
| 35 | Jasmone | 1404 | 1392 | 0.05 ± 0.00 | 0.03 ± 0.00 | 0.03 ± 0.01 | 0.01 ± 0.00 | OT |
| 36 | *α*-Gurjunene | 1410 | 1409 | 0.30 ± 0.01 | 0.16 ± 0.00 | 0.16 ± 0.00 | 0.24 ± 0.01 | BS |
| 37 | *β*-Caryophyllene | 1420 | 1419 | 1.02 ± 0.06 | 0.67 ± 0.07 | 0.58 ± 0.04 | 0.83 ± 0.04 | BS |
| 38 | *β*-Gurjunene | 1430 | 1433 | 0.17 ± 0.04 | 0.11 ± 0.04 | 0.10 ± 0.02 | 0.12 ± 0.03 | BS |
| 39 | Aromadendrene | 1446 | 1441 | 0.08 ± 0.02 | 0.04 ± 0.01 | 0.05 ± 0.01 | 0.12 ± 0.01 | BS |
| 40 | *α*-Humulen | 1455 | 1454 | 0.12 ± 0.02 | 0.08 ± 0.02 | 0.07 ± 0.01 | 0.09 ± 0.02 | MS |
| 41 | 9-epi-*β*-Caryophyllene | 1460 | 1466 | 0.79 ± 0.02 | 0.41 ± 0.01 | 0.36 ± 0.01 | 0.51 ± 0.02 | BS |
| 42 | Germacrene D | 1483 | 1485 | 3.65 ± 0.18 | 3.57 ± 0.18 | 2.93 ± 0.11 | 4.55 ± 0.09 | BS |
| 43 | Guaiene <*cis*-*β*-> | 1496 | 1493 | t | t | 0.07 ± 0.00 | 0.35 ± 0.01 | BS |
| 44 | Bicyclogermacrene | 1498 | 1500 | 1.09 ± 0.10 | 0.77 ± 0.09 | 0.60 ± 0.05 | 0.99 ± 0.06 | BS |
| 45 | *α*-Muurolene | 1501 | 1500 | 0.02 ± 0.00 | t | t | t | BS |
| 46 | Guaiene <*trans*-*β*-> | 1506 | 1502 | 0.03 ± 0.02 | t | t | t | BS |
| 47 | *δ*-Cadinene | 1526 | 1523 | 0.20 ± 0.01 | 0.19 ± 0.01 | 0.16 ± 0.00 | 0.23 ± 0.02 | BS |
| 48 | Spathulenol | 1579 |  | 0.15 ± 0.01 | 0.05 ± 0.01 | 0.05 ± 0.00 | 0.08 ± 0.00 | BSO |
| 49 | Viridiflorol | 1594 | 1592 | 0.86 ± 0.01 | 1.17 ± 0.01 | 0.77 ± 0.01 | 0.89 ± 0.01 | BSO |
| 50 | Selina-3,11-dien-6-a-ol | 1642 | 1644 | 0.04 ± 0.00 | t | t | t | BSO |
| 51 | Cadinol-epi-*α* | 1646 | 1640 | t | 0.11 ± 0.01 | 0.1 ± 0.01 | 0.1 ± 0.00 | BSO |
| 52 | Himachalol | 1651 | 1653 | 0.01 ± 0.00 | t | t | t | BSO |
| 53 | *α*-Cadinol | 1659 | 1654 | 0.12 ± 0.00 | 0.17 ± 0.01 | 0.14 ± 0.01 | 0.17 ± 0.00 | BSO |
| 54 | Cedren-13-ol <8> | 1688 | 1689 | t | 0.00 ± 0.01 | 0.01 ± 0.00 | 0.10 ± 0.01 | BSO |
| 55 | Eudesm-7(11)-en-4-ol | 1693 | 1700 | 0.13 ± 0.04 | t | t | t | BSO |
| 56 | Himachalen-ol | 1694 | 1699 | t | 0.09 ± 0.03 | 0.08 ± 0.02 | 0.12 ± 0.02 | BSO |
| 57 | Eptadecane | 1710 | 1700 | 0.02 ± 0.00 | 0.02 ± 0.00 | 0.06 ± 0.00 | 0.28 ± 0.01 | OT |
| 58 | Others |  |  | 0.91 ± 0.01 | 0.68 ± 0.05 | 0.83 ± 0.01 | 3.23 ± 0.04 |  |

Abbreviations: SE: standard error; Exp. RI: experimental retention index; Ref. RI: retention indices reported in literature (Adams, 2017); t: traces; AM: aliphatic monoterpenes; MM: monocyclic monoterpenes; BM: bi-and tri-cyclic monoterpenes; AMO: aliphatic monoterpenoids; MMO: monocyclic monoterpenoids; BMO: bi- and tri-cyclic monoterpenoids; AS: aliphatic sesquiterpenes; MS: monocyclic sesquiterpenes; BS: bi- and tri-cyclic sesquiterpenes; ASO: aliphatic sesquiterpenoids; MSO: monocyclic sesquiterpenoids; BSO: bi- and tri-cyclic sesquiterpenoids; OT: others.

**Supplementary Table 2.** Chemical composition and chromatogram peak area of the essential oils extracted from *O. basilicum* grown under different LTs. The compounds are listed according to their elution on a Rtx®-5 Restek capillary column.

| **No.** | **Compound** | **Exp. RI** | **Ref. RI** | **SUN**  **(area % ± SE)** | **HPS200**  **(area % ± SE)** | **HPS100**  **(area % ± SE)** | **CoeLux®**  **(area % ± SE)** | **Abbr.** |
| --- | --- | --- | --- | --- | --- | --- | --- | --- |
| 1 | *α*-pinene | 936 | 939 | 0.17 ± 0.01 | 0.25 ± 0.02 | 0.36 ± 0.01 | 0.18 ± 0.00 | BM |
| 2 | Camphene | 951 | 954 | 0.02 ± 0.00 | 0.04 ± 0.00 | 0.06 ± 0.01 | 0.04 ± 0.00 | BM |
| 3 | Sabinene | 976 | 975 | 0.13 ± 0.02 | 0.26 ± 0.03 | 0.33 ± 0.03 | 0.17 ± 0.01 | BM |
| 4 | *β*-pinene | 977 | 979 | 0.30 ± 0.01 | 0.60 ± 0.01 | 0.67 ± 0.03 | 0.38 ± 0.00 | BM |
| 5 | Myrcene | 993 | - | 0.34 ± 0.00 | 0.61 ± 0.01 | 0.65 ± 0.02 | 0.35 ± 0.00 | AM |
| 6 | *α*-terpinen | 1016 | 1017 | 0.05 ± 0.00 | 0.06 ± 0.00 | 0.07 ± 0.01 | 0.09 ± 0.00 | MM |
| 7 | Limonene | 1030 | 1029 | 0.22 ± 0.00 | 0.28 ± 0.02 | 0.39 ± 0.02 | 0.16 ± 0.01 | MM |
| 8 | 1,8-cineole (eucalyptol) | 1034 | 1031 | 2.74 ± 0.02 | 6.70 ± 0.04 | 5.36 ± 0.03 | 4.28 ± 0.04 | BMO |
| 9 | Ocimene <Z-*β*-> | 1042 | 1037 | 0.02 ± 0.00 | 0.05 ± 0.01 | 0.09 ± 0.01 | 0.09 ± 0.00 | AM |
| 10 | Ocimene <E-*β*-> | 1053 | 1050 | 0.62 ± 0.00 | 1.12 ± 0.03 | 1.13 ± 0.02 | 0.87 ± 0.00 | AM |
| 11 | *γ*-terpinene | 1061 | 1059 | 0.11 ± 0.00 | 0.12 ± 0.00 | 0.14 ± 0.01 | 0.19 ± 0.00 | MM |
| 12 | Terpinolen | 1089 | 1088 | 0.16 ± 0.00 | 0.15 ± 0.01 | 0.22 ± 0.01 | 0.26 ± 0.00 | MM |
| 13 | Linalool | 1104 | 1096 | 25.70 ± 0.37 | 25.76 ± 0.07 | 21.65 ± 0.09 | 15.12 ± 0.16 | AMO |
| 14 | Camphor | 1148 | 1146 | 0.25 ± 0.01 | 0.25 ± 0.00 | 0.35 ± 0.00 | 0.44 ± 0.00 | BMO |
| 15 | Menthone | 1158 | 1152 | 0.14 ± 0.00 | t | t | t | MMO |
| 16 | Borneol | 1169 | 1169 | 0.30 ± 0.01 | 0.35 ± 0.01 | 0.59 ± 0.01 | 0.90 ± 0.02 | BMO |
| 17 | Menthol | 1176 | 1171 | 0.27 ± 0.01 | t | 0.05 ± 0.01 | t | MMO |
| 18 | Terpinen-4-ol | 1180 | 1177 | 0.28 ± 0.01 | 0.29 ± 0.01 | 0.39 ± 0.00 | 0.73 ± 0.02 | BMO |
| 19 | *α*-terpineol | 1194 | 1188 | 0.60 ± 0.07 | 0.76 ± 0.04 | 0.54 ± 0.02 | t | MMO |
| 20 | Estragole (methyl chavicol) | 1199 | 1196 | 22.31 ± 0.13 | 16.61 ± 0.05 | 25.22 ± 0.12 | 30.69 ± 0.19 | OT |
| 21 | Chavicol | 1260 | 1250 | 0.37 ± 0.01 | 0.77 ± 0.02 | 0.27 ± 0.00 | 0.37 ± 0.01 | OT |
| 22 | Bornyl acetate | 1289 | 1288 | 1.02 ± 0.00 | 0.62 ± 0.01 | 0.64 ± 0.01 | 0.69 ± 0.01 | BMO |
| 23 | *δ*-elemene | 1339 | 1338 | 0.31 ± 0.13 | 0.16 ± 0.04 | 0.20 ± 0.02 | 0.33 ± 0.00 | MS |
| 24 | Eugenol | 1365 | 1359 | 11.59 ± 0.08 | 26.71 ± 0.11 | 21.27 ± 0.09 | 22.76 ± 0.10 | OT |
| 25 | *β*-elemene | 1394 | 1390 | 1.19 ± 0.31 | 0.75 ± 0.06 | 0.98 ± 0.01 | 1.2 ± 0.02 | MS |
| 26 | Methyl eugenol | 1409 | 1403 | 0.69 ± 0.01 | 0.78 ± 0.02 | 2.32 ± 0.01 | 1.53 ± 0.00 | OT |
| 27 | *β*-Ylangene | 1420 | 1420 | 0.42 ± 0.04 | 0.20 ± 0.03 | 0.23 ± 0.01 | 0.34 ± 0.01 | BS |
| 28 | *β*-copaene | 1430 | 1432 | 0.08 ± 0.04 | 0.06 ± 0.02 | 0.06 ± 0.01 | 0.10 ± 0.00 | BS |
| 29 | *α-trans-*Bergamotene | 1439 | 1434 | 4.13 ± 0.06 | 3.15 ± 0.01 | 2.73 ± 0.02 | 2.93 ± 0.15 | BS |
| 30 | *α*-Guaiene | 1441 | 1439 | 0.20 ± 0.04 | 0.27 ± 0.03 | 0.36 ± 0.02 | 0.29 ± 0.07 | BS |
| 31 | Muurola-3.5-diene <*cis*> | 1448 | 1450 | 0.38 ± 0.04 | 0.21 ± 0.01 | 0.19 ± 0.01 | 0.22 ± 0.00 | BS |
| 32 | *α*-Humulene | 1455 | 1454 | 0.67 ± 0.03 | 0.29 ± 0.01 | 0.35 ± 0.00 | 0.39 ± 0.01 | MS |
| 33 | Aromadendrane <*dehydro*> | 1459 | 1462 | 0.45 ± 0.02 | 0.21 ± 0.00 | 0.33 ± 0.01 | 0.37 ± 0.01 | BS |
| 34 | Cadin-1(6).4-diene<*cis*> | 1464 | 1463 | 0.76 ± 0.04 | 0.47 ± 0.01 | 0.44 ± 0.01 | 0.47 ± 0.02 | BS |
| 35 | Germacrene D | 1483 | 1485 | 2.53 ± 0.05 | 1.72 ± 0.09 | 1.68 ± 0.05 | 1.69 ± 0.11 | MS |
| 36 | *α*-Amorphene | 1486 | 1484 | 0.27 ± 0.02 | 0.16 ± 0.01 | 0.13 ± 0.01 | 0.18 ± 0.03 | BS |
| 37 | *γ*-Amorphene | 1497 | 1495 | 1.32 ± 0.07 | 0.53 ± 0.03 | 0.56 ± 0.02 | 0.80 ± 0.01 | BS |
| 38 | Guaiene <*trans-β-*> | 1507 | 1502 | 0.94 ± 0.17 | 0.57 ± 0.04 | 0.65 ± 0.03 | 0.79 ± 0.01 | BS |
| 39 | *γ*-Cadinene | 1517 | 1513 | 3.00 ± 0.02 | 1.81 ± 0.02 | 1.59 ± 0.01 | 1.77 ± 0.02 | BS |
| 40 | *δ*-Cadinene | 1526 | 1523 | 0.66 ± 0.02 | 0.35 ± 0.00 | 0.32 ± 0.00 | 0.43 ± 0.01 | BS |
| 41 | Germacrene B | 1566 | 1561 | 0.11 ± 0.01 | 0.11 ± 0.00 | 0.11 ± 0.00 | 0.08 ± 0.00 | MS |
| 42 | Spathulenol | 1580 | 1578 | 0.11 ± 0.01 | 0.05 ± 0.00 | t | t | BSO |
| 43 | Globulol | 1586 | 1590 | 0.12 ± 0.01 | 0.04 ± 0.00 | t | 0.03 ± 0.00 | BSO |
| 44 | Cubenol <1.10-di-epi-> | 1618 | 1619 | 1.39 ± 0.06 | 0.55 ± 0.01 | 0.59 ± 0.01 | 0.72 ± 0.00 | BSO |
| 45 | Cubenol | 1649 | 1646 | 10.33 ± 0.08 | 4.19 ± 0.05 | 4.44 ± 0.03 | 5.00 ± 0.21 | BSO |
| 46 | *α*-eudesmol | 1656 | 1653 | 0.24 ± 0.01 | 0.12 ± 0.01 | 0.10 ± 0.01 | 0.03 ± 0.00 | BSO |
| 47 | *α*-Cadinol | 1660 | 1652 | 0.55 ± 0.03 | 0.16 ± 0.01 | 0.17 ± 0.00 | 0.23 ± 0.00 | BSO |
| 48 | Others |  |  | 1.43 ± 0.04 | 0.76 ± 0.00 | 1.08 ± 0.02 | 1.33 ± 0.01 |  |

Abbreviations: SE: standard error; Exp. RI: experimental retention index; Ref. RI: retention indices reported in literature (Adams, 2017); t: traces; AM: aliphatic monoterpenes; MM: monocyclic monoterpenes; BM: bi-and tri-cyclic monoterpenes; AMO: aliphatic monoterpenoids; MMO: monocyclic monoterpenoids; BMO: bi- and tri-cyclic monoterpenoids; AS: aliphatic sesquiterpenes; MS: monocyclic sesquiterpenes; BS: bi- and tri-cyclic sesquiterpenes; ASO: aliphatic sesquiterpenoids; MSO: monocyclic sesquiterpenoids; BSO: bi- and tri-cyclic sesquiterpenoids; OT: others.

**Supplementary Table 3.** Raw data presented in figures 5, 6, and 7 relative to the morphological and anatomical traits measured in *Mentha piperita* and *O. basilicum* plants grown under different LTs

| **Plant  species** | **Treatment** | **Shoot biomass (g)** | **Root biomass (g)** | **Shoot**  **to**  **Root**  **ratio** | **Lamina**  **to**  **Petiole**  **ratio** | **Leaf area (cm2)** | **Leaves biomass (g)** | **Leaf mass per area (g m^-2^)** | **Whole leaf thickness (µm)** | **Palisade thickness (µm)** | **Leaf**  **to**  **Palisade**  **ratio** |
| --- | --- | --- | --- | --- | --- | --- | --- | --- | --- | --- | --- |
|  |  |  |  |  |  |  |  |  |  |  |  |
| ***Ocimum basilicum*** | **HPS200** | 5,901 | 1,319 | 4,475 | 9,345 | 478,262 | 2,526 | 52,813 | 267,385 | 80,413 | 3,325 |
|  |  | 8,631 | 1,467 | 5,883 | 7,404 | 363,018 | 3,374 | 92,947 | 222,448 | 71,165 | 3,126 |
|  |  | 5,242 | 1,317 | 3,979 | 4,598 | 612,144 | 2,639 | 43,106 | 255,810 | 72,340 | 3,536 |
|  |  | 9,707 | 1,562 | 6,215 | 5,803 | 547,720 | 4,130 | 75,399 | 220,298 | 71,938 | 3,062 |
|  |  | 5,698 | 1,248 | 4,565 | 5,045 | 402,761 | 3,033 | 75,315 | 240,723 | 72,292 | 3,330 |
|  |  | 8,366 | 2,350 | 3,560 | 6,393 | 364,162 | 4,336 | 119,080 | 292,493 | 83,552 | 3,501 |
|  |  |  |  |  |  |  |  |  |  |  |  |
|  | **HPS100** | 9,216 | 0,962 | 9,584 | 5,078 | 773,619 | 5,438 | 63,710 | 230,408 | 70,195 | 3,282 |
|  |  | 6,657 | 0,963 | 6,912 | 4,010 | 631,620 | 4,269 | 47,715 | 198,858 | 53,233 | 3,736 |
|  |  | 5,463 | 0,683 | 7,994 | 4,888 | 783,678 | 3,179 | 52,265 | 205,793 | 62,162 | 3,311 |
|  |  | 6,109 | 0,728 | 8,395 | 5,664 | 707,906 | 3,823 | 71,530 | 202,637 | 62,886 | 3,222 |
|  |  | 7,950 | 1,188 | 6,690 | 6,697 | 824,723 | 3,951 | 40,724 | 203,332 | 69,110 | 2,942 |
|  |  | 4,684 | 0,606 | 7,734 | 4,732 | 739,675 | 3,143 | 41,442 | 234,590 | 73,997 | 3,170 |
|  |  |  |  |  |  |  |  |  |  |  |  |
|  | **CoeLux®** | 3,450 | 0,412 | 8,367 | 4,997 | 853,481 | 1,966 | 31,475 | 195,733 | 60,513 | 3,235 |
|  |  | 3,025 | 0,435 | 6,958 | 9,650 | 894,777 | 1,487 | 23,875 | 177,173 | 64,040 | 2,767 |
|  |  | 2,637 | 0,289 | 9,121 | 5,391 | 608,273 | 1,782 | 31,257 | 207,057 | 62,180 | 3,330 |
|  |  | 3,427 | 0,466 | 7,353 | 6,720 | 534,529 | 1,913 | 30,262 | 155,218 | 55,250 | 2,809 |
|  |  | 3,434 | 0,446 | 7,695 | 3,612 | 970,223 | 1,988 | 21,476 | 187,160 | 68,135 | 2,747 |
|  |  | 2,571 | 0,330 | 7,781 | 4,889 | 758,529 | 1,643 | 24,793 | 188,910 | 70,875 | 2,665 |
|  |  |  |  |  |  |  |  |  |  |  |  |
| ***Mentha piperita*** | **HPS200** | 8,136 | 2,087 | 3,899 | 8,452 | 977,124 | 3,543 | 45,793 | 175,897 | 83,053 | 2,118 |
|  |  | 8,061 | 1,741 | 4,630 | 9,348 | 1051,349 | 3,752 | 59,405 | 189,503 | 78,858 | 2,403 |
|  |  | 6,175 | 1,430 | 4,319 | 9,136 | 1073,108 | 2,807 | 35,813 | 186,920 | 81,732 | 2,287 |
|  |  | 9,148 | 1,649 | 5,549 | 8,187 | 1041,993 | 4,290 | 60,607 | 200,043 | 92,132 | 2,171 |
|  |  | 8,676 | 1,739 | 4,990 | 7,133 | 1131,828 | 3,470 | 42,075 | 179,122 | 79,817 | 2,244 |
|  |  | 8,795 | 1,686 | 5,218 | 9,541 | 950,491 | 4,509 | 60,965 | 157,237 | 75,873 | 2,072 |
|  |  |  |  |  |  |  |  |  |  |  |  |
|  | **HPS100** | 8,859 | 1,288 | 6,878 | 7,746 | 624,708 | 3,686 | 37,724 | 152,675 | 59,752 | 2,555 |
|  |  | 7,422 | 1,195 | 6,210 | 10,656 | 622,633 | 3,561 | 33,870 | 157,033 | 66,882 | 2,348 |
|  |  | 6,390 | 0,884 | 7,230 | 6,002 | 570,022 | 2,984 | 27,809 | 138,473 | 55,760 | 2,483 |
|  |  | 7,210 | 0,906 | 7,957 | 7,033 | 632,279 | 3,506 | 33,649 | 149,382 | 68,532 | 2,180 |
|  |  | 6,079 | 0,900 | 6,756 | 8,320 | 925,477 | 2,960 | 26,149 | 163,140 | 71,463 | 2,283 |
|  |  | 5,901 | 0,650 | 9,075 | 8,111 | 662,576 | 3,149 | 33,131 | 148,157 | 59,627 | 2,485 |
|  |  |  |  |  |  |  |  |  |  |  |  |
|  | **CoeLux®** | 6,036 | 0,585 | 10,323 | 5,601 | 827,858 | 2,819 | 34,052 | 126,548 | 53,343 | 2,372 |
|  |  | 3,013 | 0,218 | 13,810 | 7,778 | 974,810 | 1,582 | 16,233 | 114,295 | 41,857 | 2,731 |
|  |  | 4,982 | 0,411 | 12,118 | 7,355 | 1047,244 | 2,337 | 22,313 | 119,455 | 45,243 | 2,640 |
|  |  | 4,181 | 0,389 | 10,741 | 6,598 | 739,447 | 1,953 | 26,411 | 133,248 | 54,870 | 2,428 |
|  |  | 2,420 | 0,232 | 10,436 | 6,601 | 1021,364 | 1,102 | 10,789 | 117,182 | 49,755 | 2,355 |
|  |  | 5,407 | 0,609 | 8,872 | 5,858 | 1012,452 | 2,399 | 23,696 | 108,252 | 46,892 | 2,309 |
|  |  |  |  |  |  |  |  |  |  |  |  |

**Supplementary Table 4.** Raw data presented in supplementary figures 1 and 2 relative to the leaf traits measured in *Mentha piperita* and *O. basilicum* plants grown under different LTs

| **Plant  species** | **Treatment** | **Peltate glands (No. cm^-2^)** | **Peltate glands (No. leaf^-1^)** | **Capitate glands (No. cm^-2^)** | **Capitate glands (No. leaf^-1^)** |
| --- | --- | --- | --- | --- | --- |
|  |  |  |  |  |  |
| ***Ocimum basilicum*** | **HPS200** | 124 | 7518 | 1072 | 65056 |
|  |  | 381 | 16203 | 2026 | 86162 |
|  |  | 114 | 8355 | 1067 | 77977 |
|  |  | 270 | 15640 | 1286 | 74522 |
|  |  | 470 | 23411 | 1417 | 70528 |
|  |  | 517 | 28311 | 2781 | 152383 |
|  |  |  |  |  |  |
|  | **HPS100** | 190 | 13649 | 1087 | 77912 |
|  |  | 63 | 6686 | 698 | 73546 |
|  |  | 365 | 24441 | 1270 | 85013 |
|  |  | 190 | 9804 | 1000 | 51471 |
|  |  | 177 | 15505 | 1000 | 87806 |
|  |  | 159 | 12246 | 849 | 65516 |
|  |  |  |  |  |  |
|  | **CoeLux®** | 643 | 39304 | 964 | 58957 |
|  |  | 214 | 14372 | 861 | 57753 |
|  |  | 286 | 18120 | 1226 | 77764 |
|  |  | 288 | 15256 | 887 | 46965 |
|  |  | 691 | 53164 | 1214 | 93496 |
|  |  | 289 | 24777 | 736 | 63134 |
|  |  |  |  |  |  |
| ***Mentha piperita*** | **HPS200** | 613 | 26782 | 1339 | 58500 |
|  |  | 890 | 35524 | 2081 | 83081 |
|  |  | 891 | 42998 | 2172 | 104817 |
|  |  | 786 | 31574 | 1448 | 58179 |
|  |  | 748 | 36051 | 2208 | 106467 |
|  |  | 776 | 33039 | 1373 | 58447 |
|  |  |  |  |  |  |
|  | **HPS100** | 668 | 40838 | 2281 | 139350 |
|  |  | 599 | 34064 | 1674 | 95164 |
|  |  | 1018 | 59291 | 1176 | 68522 |
|  |  | 433 | 24221 | 1005 | 56141 |
|  |  | 839 | 48353 | 1403 | 80804 |
|  |  | 875 | 44604 | 1192 | 60733 |
|  |  |  |  |  |  |
|  | **CoeLux®** | 1083 | 55857 | 1765 | 90977 |
|  |  | 567 | 32812 | 1222 | 70733 |
|  |  | 1030 | 60875 | 2036 | 120356 |
|  |  | 552 | 24470 | 1041 | 46097 |
|  |  | 1054 | 61550 | 2323 | 135682 |
|  |  | 917 | 50307 | 1312 | 72006 |
|  |  |  |  |  |  |
